# Steroidal Saponins from the Rhizomes of *Aspidistra typica*

**DOI:** 10.1371/journal.pone.0150595

**Published:** 2016-03-03

**Authors:** Jiang-Ming Cui, Li-Ping Kang, Yang Zhao, Jian-Yuan Zhao, Jie Zhang, Xu Pang, He-Shui Yu, De-Xian Jia, Chao Liu, Li-Yan Yu, Bai-Ping Ma

**Affiliations:** 1 Beijing Institute of Radiation Medicine, Beijing, 100850, PR China; 2 School of Pharmaceutical Sciences, Central South University, Changsha, 410013, China; 3 State Key Laboratory Breeding Base of Dao-di Herbs, National Resource Center for Chinese Materia Medica, China Academy of Chinese Medical Sciences, Beijing, 100700, People’s Republic of China; 4 Institute of Medicinal Biotechnology, Chinese Academy of Medical Sciences & Peking Union Medical College, Beijing, 100050, China; 5 Ovation Health Science and Technology Co. Ltd., ENN Group, Langfang, 065001, China; National Cancer Institute at Frederick, UNITED STATES

## Abstract

Eleven new furostanol saponins, typaspidosides B-L (**1**–**11**), one new spirostanol saponin, typaspidoside M (**12**), and five known spirostanol saponins, 25*S*-atropuroside (**13**), neoaspidistrin (**14**), (25*S*)-pratioside D_1_ (**15**), 25*S*-aspidistrin (**16**) and 25*S*-neosibiricoside (**17**) were isolated from the rhizomes of *Aspidistra typica* Baill. The structures of the new compounds were established using 1D and 2D NMR (^1^H-^1^H COSY, HMQC, HMBC and ROESY) spectroscopy, high resolution mass spectrometry, and chemical methods. The aglycones of **1**–**3** (unusual furostanol saponins with opened E ring type), **9** and **10** (the methoxyl substituent at C-23 position) were found, identified from natural products for the first time. Moreover, the anti-HIV activities of the isolated steroidal glycosides were assessed, and compounds **13**, **14**, **16** and **17** exhibited high active against HIV-1.

## Introduction

**Fig 1:**
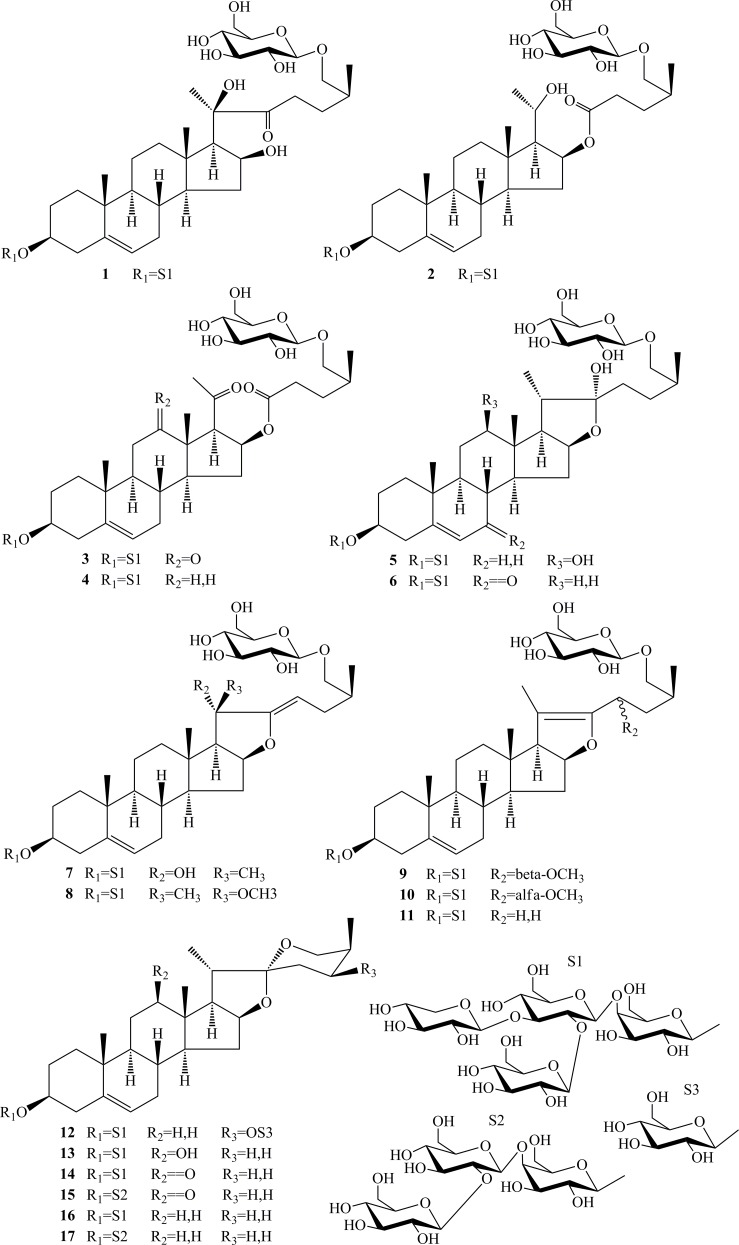
The structures of compounds 1–17.

*Aspidistra typica* Baill is a perennial herbaceous plant which belongs to the family Convallariaceae [1]. The rhizomes of *A*. *typica*, a kind of folk herbal medicine termed “Zhi Zhu Bao Dan”, are used to treat fractures, congestion and snake-bite in southwestern China and northern Vietnam [2]. A wide range of steroidal saponins have been extracted from the family Convallariaceae, but no more than 30 compounds were reported from the genus *Aspidistra* [3–7]. In order to characterize the chemical composition and active steroidal saponins of *A*. *typical*, systemic phytochemical analysis on the ethanol extract of the rhizomes of *A*. *typica* was carried out, which led to the isolation and identification of twelve new steroidal saponins, including eleven furostanol saponins (**1**–**11**) and one spirostanol saponin (**12**), along with five known spirostanol saponins, 25*S*-atropuroside H (**13**) [8], neoaspidistrin (**14**) [9], (25*S*)-pratioside D_1_ (**15**) [10,11], 25*S*-aspidistrin (**16**) [2,3] and 25*S*-neosibiricoside D (**17**) [12,13] (Fig 1). The structures of these new compounds were elucidated mainly by NMR spectroscopic and mass spectroscopic methods, and the known compounds were identified by comparing their NMR and MS data with those in the literatures. Among these compounds, the aglycones of **1**–**3**, **9** and **10** were found, identified for the first time, while **13** and **15** were firstly isolated as monomers from natural products. The all 17 compounds showed different degrees for anti-HIV activities. Compounds **14 (**99.95±0.01) and **16** (99.95±0.02) showed the highest activity inhibition of viral replication.

## Material and Methods

### General

The HRESIMS spectra were recorded on a SYNAPT Q-TOF MS (Waters Corporation, Milford, MA, USA). The NMR spectra were recorded on a Varian ^UNITY^INOVA 600 spectrometer (600 MHz for ^1^H NMR and 150 MHz for ^13^C-NMR, Paloalto, USA) in pyridine-*d*_5_, (^1^H, *δ*_H_ 7.21, 7.58, 8.74; ^13^C, *δ*_C_ 123.5, 136.0, 150.3) and 2D NMR experiments were performed using standard microprograms. Optical rotations were measured with a Perkin-Elmer 343 polarimeter (PerkinElmer, Waltham, MA, USA). HPLC was performed on a Waters 2695 series; XBP C_18_ and XBP C_18_-2 columns (5 μm, 4.6 × 250 mm, Agela, Tianjin, China); Alltech 2000 ELSD detector (Alltech Corporation, Deerfield, USA). TLC was performed on silica gel GF254 plates (Qingdao marine Chemical, China). Column chromatography (CC) was performed on macroporous resin SP825 (Mitsubishi Chemicals, Japan), silica gel (Qingdao Haiyang Chemical Co., Ltd, China), MCI gel (50 μm, Mitsubishi Chemicals, Tokyo, Japan)) and ODS-A silica gel (120 Å, 50 μm, YMC, Japan).

### Plant material

The rhizomes of Aspidistra typica Baill were collected from Jinghong region of the Yunnan Province, People's Republic of China, in November 2010. The plant was identified by Prof. Li-xia Zhang (Institute of Medicinal Plant Development Yunnan Branch). A voucher specimen (No. 100802) was deposited in the Herbarium of the Beijing Institute of Radiation Medicine, Beijing. No specific permits were required for the described field study, as no endangered or protected species were sampled, and the locality where the sample came from is not protected in any way.

### Extraction and isolation

The air-dried rhizomes of *A*. *typica* (4 kg) were refluxed in EtOH-H_2_O (6:4, v/v, 32 L × 2). The combined extract was evaporated under reduced pressure. The concentrated suspended in water and partitioned with n-BuOH (1:1, v/v) to give an n-BuOH -soluble fraction (183.0 g). The n-BuOH fraction was subjected to a silica-gel chromatography with a gradient mixture of CHCl_3_-MeOH (3:1, 2:1, 2:1 and 1:2, v/v) as the eluent, and four fractions were obtained (A−B). Fr. A (59.0) was chromatographed on a MCI gel column and eluted with a gradient mixture of EtOH-H_2_O (1:3, 8:17, 2:3, 1:1 and 3:2 (v/v) to yield seven factions (A1–A7). Fr. A1 (9.2 g) was subjected to ODS silica-gel CC eluted with Me_2_CO-H_2_O (2:8, v/v) and a total of 243 tubes were collected. Tubes 187–198 was repeatedly by CC on ODS silica-gel (eluted with MeCN-H_2_O, 2:8, v/v) and preparative HPLC (eluted with MeCN-H_2_O, 11:39, v/v) to yield **6** (11.9 mg). Fr. A2 (1.1 g) was subjected to ODS silica-gel CC eluted with MeOH-H_2_O (9:11, v/v) and a total of 110 tubes were collected. Tubes 42–59 was purified by semi-preparative HPLC with MeCN-H_2_O (19:81, v/v) to yield **3** (10.6 mg). Tubes 50–67 was purified by semi-preparative HPLC with MeOH-H_2_O (9:11, v/v) to yield **5** (38.1 mg). Fr. A3 (0.25 g) was subjected to ODS silica-gel CC eluted with MeOH-H_2_O (9:11, v/v) and a total of 110 tubes were collected. Tubes 42–59 was purified by semi-preparative HPLC with MeCN-H_2_O (19:81, v/v) to yield **3** (10.6 mg). Tubes 50–67 was purified by semi-preparative HPLC with MeOH-H_2_O (9:11, v/v) to yield **5** (38.1 mg). Fr. A4 (0.28 g) was subjected to ODS silica-gel CC eluted with MeOH-H_2_O (9:11, v/v) and a total of 50 tubes were collected. Tubes 13–20 was purified by semi-preparative HPLC with MeCN-H_2_O (21:79, v/v) to yield **1** (22.6 mg) and **2** (7.5 mg). Tubes 30–34 was evaporated under reduced pressure to yield **7** (20.6 mg). Fr. A5 (8.0 g) was subjected to ODS silica-gel CC eluted with Me_2_CO-H_2_O (7:13, v/v) and a total of 135 tubes were collected. Tubes 125–126 was further separated by preparative HPLC, eluted with MeCN-H_2_O (29:71, v/v) to yield **6** (34.1 mg) and **12** (32.3 mg). Tube 129 was purified by semi-preparative HPLC with Me_2_CO-H_2_O (16:84) to yield **8** (34.4 mg) and **10** (52.3 mg). Fr. A6 (0.12 g) was separated by preparative HPLC, eluted with MeOH-H_2_O (29:71, v/v) to yield **4** (35.1 mg). Fr. A7 (0.04 g) was purified by preparative HPLC with MeOH-H_2_O (67:33, v/v) to yield **5** (18.1 mg). Fr. B (2.1) was separated CC on a silica-gel with CHCl_3_-MeOH-H_2_O (3513:2, v/v, low layer) to give 50 tubes. Tubes 19–25 was subjected to ODS silica-gel CC eluted with MeOH-H_2_O (7:3, v/v) to yield **13** (14.1 mg). Fr. C (15.5) was separated CC on a silica-gel with CHCl_3_-MeOH (7:1, v/v) to give four subfractions (C1-C4). Subfraction C4 was subjected to ODS silica-gel CC eluted with MeOH-H_2_O (7:3, v/v) to yield **15** (55.0 mg). Fr. D (2.6 g) was recrystallized from MeOH and filtered to afford **16** (54.1 g). The mother liquid was concentrated under reduced pressure and chromatographed on ODS silica-gel with MeOH-H_2_O (3:1) to provide three subfractions (D1–D3). Subfraction D1 was further purified by semi-preparative HPLC with MeOH-H_2_O (79:21) to yield **17** (15.6 mg), and D2 was further separated by semi-preparative HPLC with MeOH-H_2_O (78:22) to yield **14** (10.5 mg).

Typaspidoside B (**1**): White amorphous power; ${\left[\alpha \right]}_{\text{D}}^{20}$ -45.0 (c 0.04, pyridine); ^1^H-NMR and ^13^C-NMR data, see Tables 1 and 2; HRESIMS (negative) *m*/*z*: 1277.5665 [M−H]^−^ (calc. for C_56_H_91_O_29_, 1227.5646).

**Table 1:** ^1^H and ^13^C NMR data of the aglycone moiety of saponins 1–6 in pyridine-*d*_5_.

Position	1		2		3		4		5		6	
	*δ*_C_	*δ*_H_ *J* (Hz)	*δ*_C_	δ_H_ J (Hz)	*δ*_C_	δ_H_ J (Hz)	*δ*_C_	*δ*_H_ *J* (Hz)	*δ*_C_	δ_H_ J (Hz)	*δ*_C_	δ_H_ J (Hz)
1	37.5	0.94 m, 1.64 m	37.5	0.92 m, 1.64 m	37.0	0.85 m, 1.40 m	37.5	0.93 m, 1.65 m	37.5	0.93 m, 1.66 o	36.5	0.94 o, 1.65 o
2	30.1	1.67 m, 2.06 m	30.1	1.67 m, 2.07 m	29.8	1.63 m, 2.03 m	30.1	1.69 m, 2.08 m	30.1	1,65 o, 2.04 o	29.8	1.70 o, 2.10 m
3	78.2	3.86 m	78.2	3.88 m	77.7	3.87 m	78.1	3.88 m	78.2	3.84 m	77.1	3.90 m
4	39.3	2.39 m, 2.63 br d (12.6)	39.3	2.41 o, 2.64 m	39.0	2.37 m, 2.66 dd (3.0, 13.2)	39.3	2.37 m, 2.64 dd (3.0, 13.2)	39.2	2.39 br t (12.0), 2.64 dd (3.0, 12.0)	39.0	2.44 t (12.0), 2.73 br d (12.0)
5	141.1	–	141.2	–	141.0	–	141.2	–	141.1	–	165.3	–
6	121.6	5.28 br s	121.6	5.30 br s	121.2	5.27 br s	121.4	5.28 br s	121.8	5.28 br s	126.1	5.72 s
7	32.0	1.46 m, 1.84 m	32.1	1.50 m, 1.85 m	31.3	1.48 m, 1.87 m	31.9	1.48 m, 1.82 m	32.2	1.44 m, 1.87 m	201.0	–
8	31.2	1.48 m	31.6	1.49 m	30.4	1.81 m	31.0	1.47 m	30.9	1.54 m	45.1	2.38 t (12.0)
9	50.4	0.83 m	50.5	0.90 m	53.1	1.31 m	50.4	0.87 m	50.1	1.28 m	50.0	1.36 m
10	37.0	–	37.0	–	37.7	–	37.0	–	37.2	–	39.0	–
11	20.8	1.37 m, 1.40 m	21.0	1.47 o	37.0	2.23 dd (5.4, 13.2),2.57 t (13.2)	20.6	1.40 o	31.5	1.67 m, 1.85 m	21.1	1.40 o
12	39.7	1.11 dt (4.8, 12.6), 1.63 o*	40.1	1.34 dt (4.8, 13.2), 2.65 m	211.9	–	38.2	1.07 m, 2.14 m	79.1	3.57 m	38.7	1.03 m, 1.69 o
13	42.5	–	43.0	–	56.8	–	42.3	–	46.6	–	41.6	–
14	54.7	0.76 m	54.3	0.89 m	54.0	1.18 m	54.1	0.75 m	55.4	1.11 m	50.0	1.48 dt (6.0, 12.0)
15	37.5	1.52 dt (4.2, 12.6), 2.22 m	36.0	1.16 m, 2.41 m	34.7	1.52 o, 2.47 m	35.5	1.25 m, 2.40 m	32.2	1.60 m, 2.06 o	34.5	1.73 dt (6.0, 13.2), 3.27 dt (6.0, 13.2)
16	72.9	4.83 o	75.4	5.33 dt (4.2 7.8)	73.4	5.62 dt (4.2, 7.8)	74.7	5.64 dt (4.2 7.8)	81.2	5.02 o	81.3	5.04 m
17	59.9	1.86 d (7.2)	62.4	1.58 m	59.0	3.27 d (7.8)	66.6	2.46 d (7.2)	63.7	2.30 dd (6.6, 8.4)	62.9	1.86 dd (6.6, 7.8)
18	15.8	1.38 s	13.3	1.18 s	13.9	1.52 s	13.8	1.19 s	11.3	1.14 s	16.6	0.88 s
19	19.4	0.87 s	19.5	0.89 s	18.9	0.90 s	19.4	0.87 s	19.4	0.90 s	17.1	0.95 s
20	82.5	–	65.2	4.45 m	204.4	–	205.5	–	41.8	2.45 m	40.7	2.21 q (6.6)
21	28.6	1.72 s	24.2	1.43 d (6.6)	30.7	2.26 s	30.5	2.12 s	15.7	1.59 d (6.6)	16.6	1.31 d (6.6)
22	215.8	–	173.1	–	173.3	–	173.3	–	110.9	–	110.7	–
23	36.0	3.14 m	32.5	2.41 o, 2.46 dd (6.6, 9.0)	32.2	2.37 m, 2.42 m	32.2	2.36 m, 2.42 m	37.3	1.99 m, 2.11 m	37.3	1.96 m, 2.04 m
24	27.7	1.60 m, 2.13 m	29.2	1.58 m, 1.99 m	29.0	1.57 m, 1.94 m	29.0	1.57 m, 1.94 m	28.4	1.67 m, 2.04 o	28.4	1.66 m, 2.05 o
25	33.7	1.97 m	33.6	1.92 m	33.5	1.86 m	33.5	1.86 m	34.5	1.92 m	34.7	1.91 m
26	75.6	3.50 dd (6.6, 9.0), 4.07 o	74.8	3.46 dd (6.6, 9.6), 3.99 o	74.7	3.44 dd (6.0, 9.6), 3.95 o	74.8	3.44 dd (6.6, 9.6), 3.95 o	75.5	3.45 dd (7.2, 9.0), 4.07 o	75.4	3.45 dd (7.2, 9.6), 4.07 o
27	17.2	1.00 d (7.2)	16.9	0.96 d (6.6)	16.9	0.91 d (6.6)	16.9	0.91 d (6.6)	17.5	1.01 d (7.2)	17.5	1.01 d (6.6)

* o = overlapped

**Table 2:** ^1^H and ^13^C NMR data of the aglycone moiety of saponins 7–12 in pyridine-*d*_5_.

Position	7		8		9		10		11		12	
	*δ*_C_	*δ*_H_ *J* (Hz)	*δ*_C_	*δ*_H_ *J* (Hz)	*δ*_C_	*δ*_H_ *J* (Hz)	*δ*_C_	*δ*_H_ *J* (Hz)	*δ*_C_	*δ*_H_ *J* (Hz)	*δ*_C_	*δ*_H_ *J* (Hz)
1	37.5	0.95 m, 1.65 m	37.5	0.94 m, 1.65 m	37.5	0.96 (m), 1.66 o	37.5	0.95 m, 1.66 m	37.5	0.95 m, 1.66 m	37.5	0.94 m, 1.64 o
2	30.1	1.67 m, 2.09 m	30.1	1.69 m, 2.09 o	30.1	1.68 o, 2.09 (m)	30.1	1.69 m, 2.09 m	30.2	1.69 m, 2.08 m	30.1	1.68 m, 2.08 o
3	78.2	3.88 m	78.2	3.87 m	78.2	3.88 (m)	78.2	3.88 m	78.2	3.88 m	78.2	3.86 m
4	39.3	2.41 m, 2.64 m	39.2	2.40 brt (12.0), 2.63 br d (12.0)	39.3	2.41 brt (11.4), 2.64 dd (3.0, 12.0)	39.3	2.41 brt (12.0), 2.64 dd (3.0, 12.0)	39.3	2.41 brt (11.4), 2.64 m	39.3	2.40 brt (12.0), 2.63 br d (12.0)
5	141.1	–	141.0	–	141.0	–	141.0	–	141.0	–	141.0	–
6	121.6	5.28 br s	121.5	5.27 br d (5.4)	121.6	5.28 br d (4.8)	121.6	5.28 br d (4.2)	121.6	5.29 br s	121.6	5.27 br d (4.8)
7	32.0	1.45 m, 1.82 m	32.0	1.45 o, 1.81 o	32.3	1.47 o, 1.83 (m)	32.3	1.47 o, 1.81 m	32.4	1.48 o, 1.84 m	32.3	1.45 m, 1.82 m
8	31.1	1.50 m	31.0	1.46 o	31.4	1.47 o	31.3	1.43 m	31.4	1.47 m	31.6	1.49 dq (4.8, 10.8)
9	50.1	0.83 m	50.0	0.81 m	50.2	0.86 o	50.2	0.85 m	50.3	0.86 m	50.3	0.84 o
10	37.0	–	37.0	–	37.0	–	37.0	–	37.0	–	37.0	–
11	20.6	1.37 o	20.5	1.36 o	21.2	1.40 (m), 1.42 (m)	21.2	1.35 m, 1.41 m	21.3	1.36 m, 1.41 m	21.1	1.34 m, 1.38 m
12	39.3	1.15 m, 1.88 m	39.3	1.10 o, 1.81 m	39.6	1.16 dt (4.8, 12.6), 1.73 o	39.6	1.13 o, 1.71 m	39.7	1.13 m, 1.71 m	39.8	1.04 m, 1.63 o
13	40.4	–	40.3	–	43.3	–	43.5	–	43.4	–	40.4	–
14	57.0	0.93 m	56.7	0.88 (m)	55.1	0.86 o	54.9	0.83 m	54.9	0.84 m	56.6	0.99 m
15	33.5	1.47 m, 2.04 m	33.5	1.39 dt (13.2, 4.8), 1.98 m	34.4	1.49 dt (13.2, 5.4), 2.10 (m)	34.5	1.44 o, 2.06 o	34.5	1.46 m, 2.08 m	32.0	1.31 o, 1.93 m
16	84.3	5.20 m	83.9	4.95 dt (4.2, 7.2)	85.1	4.82 o	84.6	4.84 o	84.5	4.79 o	81.4	4.43 o
17	67.9	2.22 d (6.0)	66.7	2.06 d (6.0)	64.8	2.48 d (10.2)	64.5	2.50 d (10.2)	64.5	2.43 d (9.6)	62.2	1.72 dd (6.6, 8.4)
18	13.6	0.89 s	13.6	0.84 s	14.6	0.75 s	14.3	0.68 s	14.2	0.70 s	16.3	0.74 s
19	19.4	0.89 s	19.4	0.88 s	19.4	0.89 s	19.4	0.88 s	19.4	0.88 s	19.4	0.86 s
20	76.7	–	82.4	–	109.0	–	109.0	–	103.5	–	42.5	1.91 m
21	21.9	1.71 s	15.3	1.37 s	11.6	1.73 s	11.6	1.73 s	11.8	1.62 s	14.7	1.09 d (7.2)
22	163.8	–	157.3	–	150.7	–	150.0	–	152.5	–	111.3	–
23	91.4	4.52 br s	96.1	4.33 t (7.2)	73.1	4.18 dd (6.0, 8.4)	73.6	4.22 m	23.7	2.16 m, 2.24 m	34.1	2.09 o, 2.10 o
24	29.7	2.13 m, 2.51 m	29.6	2.19 dt (13.8, 7.8), 2.48 dt (6.0, 13.8)	37.6	1.63 m, 2.29 ddd (6.0, 8.4, 13.8)	37.3	1.86 dt (13.8, 7.8), 2.06 o	31.4	1.45 m, 1.85 m	72.9	4.80 dt (10.8, 5.4)
25	34.9	2.09 m	34.9	2.11 (m)	31.0	2.22 m	31.1	2.22 m	33.8	1.94 m	31.8	2.25 m
26	75.4	3.52 dd (6.0, 9.0), 4.16 o	75.4	3.56 dd (7.2, 9.6), 4.15 o	75.6	3.53 dd (6.6, 9.6), 4.09 o	75.4	3.55 dd (7.2, 9.6),4.14 o	75.3	3.47 dd (6.6, 9.6), 4.06 o	64.2	3.50 br d (10.2), 3.89 m
27	17.5	1.07 d (6.0)	17.6	1.10 d (6.6)	17.5	1.07 d (6.6)	17.9	1.12 d (6.6)	17.2	1.02 d (6.6)	9.9	1.31 d (6.6)
OCH_3_	–	–	48.9	3.17 s	56.3	3.35 s	55.9	3.31 s	–	–	–	–

Typaspidoside C (**2**): White amorphous power; ${\left[\alpha \right]}_{\text{D}}^{20}$ -47.0 (c 0.05, pyridine); ^1^H-NMR and ^13^C-NMR data, see Tables 1 and 2; HRESIMS (negative) *m*/*z*: 1277.5659 [M−H]^−^ (calc. for C_56_H_91_O_29_, 1227.5646).

Typaspidoside D (**3**): White amorphous power; ${\left[\alpha \right]}_{\text{D}}^{20}$ -13.0 (c 0.05, pyridine); ^1^H-NMR and ^13^C-NMR data, see Tables 1 and 2; HRESIMS (negative) *m*/*z*: 1239.5238 [M−H]^−^ (calc. for C_56_H_87_O_30_, 1239.5258).

Typaspidoside E (**4**): White amorphous power; ${\left[\alpha \right]}_{\text{D}}^{20}$ -50.0 (c 0.06, pyridine); ^1^H-NMR and ^13^C-NMR data, see Tables 1 and 2; HRESIMS (negative) *m*/*z*: 1225.5524 [M−H]^−^ (calc. for C_56_H_89_O_29_, 1225.5490).

Typaspidoside F (**5**): White amorphous power; ${\left[\alpha \right]}_{\text{D}}^{20}$ -85.0 (c 0.06, pyridine); ^1^H-NMR and ^13^C-NMR data, see Tables 1 and 2; HRESIMS (negative) *m*/*z*: 1277.5681 [M−H]^−^ (calc. for C_56_H_91_O_29_, 1227.5646).

Typaspidoside G (**6**): White amorphous power; ${\left[\alpha \right]}_{\text{D}}^{20}$ -71.1 (c 0.05, pyridine); ^1^H-NMR and ^13^C-NMR data, see Tables 1 and 2; HRESIMS (negative) *m*/*z*: 1225.5472 [M−H]^−^ (calc. for C_56_H_89_O_29_, 1225.5490).

Typaspidoside H (**7**): White amorphous power; ${\left[\alpha \right]}_{\text{D}}^{20}$ -48.3 (c 0.06, pyridine); ^1^H-NMR and ^13^C-NMR data, see Tables 3 and 4; HRESIMS (negative) *m*/*z*: 1209.5521 [M−H]^−^ (calc. for C_56_H_89_O_28_, 1209.5540).

**Table 3:** ^1^H and ^13^C NMR data of the sugar moiety of saponins 1–6 in pyridine-*d*_5_.

Position	1		2		3		4		5		6	
	*δ*_C_	*δ*_H_ (*J* Hz)	*δ*_C_	*δ*_H_ (*J* Hz)	*δ*_C_	δ_H_ (J Hz)	*δ*_C_	*δ*_H_ (*J* Hz)	*δ*_C_	*δ*_H_ (*J* Hz)	*δ*_C_	*δ*_H_ (*J* Hz)
3-*O*-Gal-1'	102.8	4.86 d (7.8)	102.7	4.86 d (7.8)	102.7	4.86 d (7.8)	102.8	4.88 d (7.8)	102.8	4.86 d (7.8)	103.0	4.86 d (7.8)
2'	73.2	4.40 o	73.2	4.40 o	73.1	4.40 o	73.2	4.42 o	73.2	4.40 o	73.1	4.40 o
3'	75.6	4.08 o	75.6	4.08 o	75.6	4.08 o	75.6	4.09 o	75.6	4.07 o	75.6	4.07 o
4'	79.9	4.58 o	79.9	4.58 o	79.9	4.58 o	79.9	4.58 o	79.9	4.58 o	79.8	4.58 o
5'	75.4	3.95 o	75.3	3.95 o	75.4	3.95 o	75.4	3.97 o	75.3	3.95 o	75.4	3.95 o
6'	60.6	4.16 o, 4.65 m	60.6	4.16 o, 4.65 m	60.6	4.16 o, 4.65 m	60.6	4.17 o, 4.67 m	60.6	4.16 o, 4.65 m	60.6	4.16 o, 4.65 m
3'-*O*-Glc-1''	105.2	5.18 d (8.4)	105.2	5.18 d (8.4)	105.2	5.18 d (8.4)	105.2	5.18 d (7.8)	105.2	5.17 d (7.8)	105.2	5.19 d (7.8)
2''	81.4	4.42 t (8.4)	81.4	4.42 t (8.4)	81.4	4.42 t (8.4)	81.4	4.42 m	81.4	4.41 t (8.4)	81.4	4.41 t (8.4)
3''	86.8	4.16 o	86.8	4.16 o	86.7	4.16 o	86.8	4.16 t (8.7)	86.8	4.15 o	86.7	4.15 o
4''	70.5	3.80 m	70.5	3.80 m	70.5	3.80 m	70.6	3.81 t (9.0)	70.5	3.80 m	70.5	3.80 m
5''	77.6	3.87 m	77.6	3.87 m	77.7	3.87 m	77.7	3.87 m	77.6	3.87 m	77.7	3.87 m
6''	63.0	4.03 o, 4.51 o	63.0	4.03 o, 4.51 o	63.0	4.03 o, 4.51 o	63.0	4.04 o, 4.51 m	63.0	4.03 o, 4.51 o	63.0	4.03 o, 4.51 o
2''-*O*-Glc-1'''	104.9	5.57 d (7.8)	104.9	5.57 d (7.8)	104.9	5.57 d (7.8)	104.9	5.57 d (7.8)	104.9	5.56 d (7.8)	104.9	5.58 d (7.8)
2'''	76.3	4.06 o	76.3	4.06 o	76.3	4.06 o	76.3	4.06 o	76.3	4.06 o	76.3	4.06 o
3'''	77.8	4.11 o	77.7	4.11 o	77.8	4.11 o	77.7	4.11 o	77.8	4.09 o	77.8	4.09 o
4'''	71.1	4.19 o	71.1	4.19 o	71.1	4.19 o	71.1	4.20 o	71.1	4.19 o	71.1	4.19 o
5'''	78.8	3.92 o	78.8	3.92 o	78.8	3.92 o	78.8	3.91 o	78.8	3.90 o	78.8	3.90 o
6'''	62.5	4.36 o, 4.56 o	62.5	4.36 o, 4.56 o	62.5	4.36 o, 4.56 o	62.5	4.37 o, 4.57 o	62.5	4.36 o, 4.56 o	62.6	4.36 o, 4.56 o
3''-*O*-Xyl-1''''	105.0	5.23 d (7.8)	105.0	5.23 d (7.8)	105.0	5.23 d (7.8)	105.0	5.23 d (7.8)	105.0	5.23 d (7.8)	105.0	5.23 d (7.8)
2''''	75.1	3.95 o	75.1	3.95 o	75.1	3.95 o	75.1	3.96 o	75.1	3.96 o	75.1	3.96 o
3''''	78.7	4.07 o	78.7	4.07 o	78.7	4.07 o	78.7	4.07 o	78.7	4.06 o	78.7	4.06 o
4''''	70.8	4.10 o	70.8	4.10 o	70.8	4.10 o	70.8	4.12 m	70.8	4.10 o	70.8	4.10 o
5''''	67.4	3.66 t (10.6), 4.21 o	67.4	3.66 t (10.6), 4.21 o	67.4	3.66 t (10.6), 4.21 o	67.4	3.66 t (10.5), 4.23 o	67.4	3.66 t (10.6), 4.21 o	67.4	3.66 t (10.6), 4.21 o
26-*O*-Glc-1'''''	105.2	4.80 d (7.8)	105.1	4.80 d (7.8)	105.0	4.80 d (7.8)	105.1	4.79 d (7.8)	105.2	4.80 d (7.8)	105.2	4.80 d (7.8)
2'''''	75.3	4.01 t (7.8)	75.2	4.01 t (7.8)	75.2	4.01 t (7.8)	75.2	4.00 o	75.3	4.01 t (7.8)	75.3	4.01 t (7.8)
3'''''	78.6	4.23 o	78.6	4.23 o	78.6	4.23 o	78.6	4.23 o	78.6	4.23 o	78.6	4.23 o
4'''''	71.7	4.22 o	71.7	4.22 o	71.7	4.22 o	71.8	4.22 o	71.7	4.22 o	71.7	4.22 o
5'''''	78.5	3.93 o	78.6	3.93 o	78.6	3.93 o	78.6	3.93 o	78.5	3.92 o	78.5	3.92 o
6'''''	62.9	4.37 o, 4.53 o	62.9	4.37 o, 4.53 o	62.9	4.37 o, 4.53 o	62.9	4.38 o, 4.55 o	62.8	4.38 o, 4.53 o	62.9	4.38 o, 4.53 o

**Table 4:** ^1^H and ^13^C NMR data of the sugar moiety of saponins 7–12 in pyridine-*d*_5_.

Position	7		8		9		10		11		12	
	*δ*_C_	*δ*_H_ (*J* Hz)	*δ*_C_	*δ*_H_ (*J* Hz)	*δ*_C_	δ_H_ (J Hz)	*δ*_C_	*δ*_H_ (*J* Hz)	*δ*_C_	δ_H_ (J Hz)	*δ*_C_	*δ*_H_ (*J* Hz)
3-*O*-Gal-1'	102.8	4.88 d (7.8)	102.8	4.88 d (7.8)	102.8	4.88 d (7.8)	102.8	4.88 d (7.8)	102.8	4.86 d (7.8)	102.8	4.88 d (7.8)
2'	73.2	4.42 o	73.2	4.41 t (8.4)	73.2	4.41 o	73.2	4.41 t (8.4)	73.2	4.41 o	73.2	4.41 o
3'	75.6	4.09 m	75.6	4.09 o	75.6	4.09 o	75.6	4.09 o	75.6	4.09 o	75.6	4.09 o
4'	79.9	4.58 o	79.9	4.58 o	79.9	4.59 br s	79.9	4.59 br s	79.9	4.59 o	79.9	4.58 br s
5'	75.3	3.96 o	75.3	3.96 o	75.4	3.96 o	75.4	3.96 o	75.4	3.95 o	75.4	3.95 o
6'	60.6	4.16 o, 4.67 t (7.8)	60.6	4.15 o, 4.66 br t (8.4)	60.6	4.16 o, 4.67 m	60.6	4.16 t (8.4), 4.66 m	60.6	4.16 o, 4.65 m	60.6	4.16 o, 4.66 m
3'-*O*-Glc-1''	105.2	5.18 d (7.8)	105.2	5.18 d (7.8)	105.2	5.18 d (7.8)	105.2	5.18 d (7.8)	105.2	5.18 d (8.4)	105.2	5.18 d (7.8)
2''	81.4	4.41 t (8.4)	81.4	4.42 o	81.4	4.42 t (8.4)	81.4	4.42 t (8.4)	81.4	4.42 t (8.4)	81.4	4.42 t (8.4)
3''	86.8	4.16 o	86.8	4.16 o	86.8	4.16 t (8.4)	86.8	4.15 o	86.8	4.16 t (8.4)	86.8	4.16 t (9.0)
4''	70.5	3.81 t (9.0)	70.5	3.81 t (9.0)	70.5	3.82 t (9.0)	70.5	3.81 t (9.0)	70.5	3.81 m	70.5	3.80 t (9.0)
5''	77.6	3.87 m	77.7	3.87 m	77.7	3.87 m	77.7	3.87 o	77.6	3.87 m	77.7	3.87 m
6''	63.0	4.04 o, 4.52 o	63.0	4.03 o, 4.51 br d (10.2)	63.0	4.04 m, 4.51 dd (4.2, 10.2)	63.0	4.04 m, 4.51 br d (10.2)	63.0	4.04 o, 4.50 m	63.0	4.04 m, 4.51 m
2''-*O*-Glc-1'''	104.9	5.57 d (7.8)	104.9	5.57 d (7.2)	104.9	5.57 d (7.8)	104.9	5.57 d (7.2)	104.9	5.57 d (7.8)	104.9	5.57 d (7.8)
2'''	76.3	4.07 o	76.3	4.06 o	76.3	4.07 o	76.3	4.06 o	76.3	4.06 o	76.3	4.06 o
3'''	77.8	4.11 o	77.8	4.09 o	77.8	4.10 o	77.8	4.10 o	77.7	4.11 o	77.8	4.10 o
4'''	71.1	4.19 o	71.1	4.20 o	71.1	4.20 m	71.1	4.19 m	71.1	4.19 o	71.1	4.20 m
5'''	78.8	3.90 m	78.8	3.91 m	78.8	3.91 m	78.8	3.91 m	78.8	3.91 o	78.8	3.91 o
6'''	62.5	4.36 m, 4.57 o	62.5	4.36 m, 4.57 o	62.5	4.36 m, 4.56 m	62.5	4.36 o, 4.57 m	62.5	4.36(o), 4.56 o	62.5	4.36 o, 4.57 br d (12.0)
3'''-*O*-Xyl-1''''	105.0	5.23 d (7.8)	105.0	5.23 d (7.8)	105.0	5.23 d (7.8)	105.0	5.23 d (7.8)	105.0	5.23 d (7.8)	105.0	5.23 d (7.8)
2''''	75.1	3.95 o	75.1	3.96 o	75.1	3.96 o	75.1	3.96 o	75.1	3.95 o	75.1	3.95 o
3''''	78.7	4.07 o	78.7	4.06 o	78.7	4.07 o	78.7	4.06 o	78.7	4.06 o	78.7	4.06 o
4''''	70.8	4.11 o	70.8	4.10 o	70.8	4.11 o	70.8	4.10 o	70.8	4.09 o	70.8	4.10 o
5''''	67.4	3.66 t (10.8), 4.22 m	67.4	3.66 t (10.2), 4.22 o	67.4	3.67 t (10.2), 4.22 o	67.4	3.66 t (10.2), 4.21 o	67.4	3.66 t (10.5), 4.21 o	67.4	3.66 t (10.8), 4.21 o
26(24)-*O*-Glc-1'''''	105.2	4.84 d (7.8)	105.2	4.86 d (7.8)	105.3	4.84 d (7.8)	105.3	4.83 d (7.8)	105.0	4.83 (d, 7.8)	101.2	5.02 d (8.4)
2'''''	75.3	4.04 o	75.3	4.03 o	75.3	4.01 (m)	75.3	4.01 t (8.4)	75.2	4.01 (t, 7.8)	75.4	4.05 o
3'''''	78.6	4.23 o	78.7	4.24 o	78.7	4.24 o	78.6	4.21 o	78.6	4.24 o	78.7	4.24 t (9.0)
4'''''	71.7	4.24 o	71.7	4.24 o	71.7	4.24 o	71.7	4.20 o	71.7	4.22 o	71.6	4.30 t (9.0)
5'''''	78.5	3.92 m	78.5	3.94 m	78.5	3.94 o	78.5	3.94 o	78.6	3.93 o	78.5	3.92 o
6'''''	62.9	4.39 m, 4.54 m	62.8	4.38 m, 4.54 dd (2.4, 12.0)	62.9	4.38 m, 4.54 m	62.9	4.36 o, 4.53 dd (2.4, 11.4)	62.9	4.37 o, 4.54 o	62.6	4.37 o, 4.47 m

Typaspidoside I (**8**): White amorphous power; ${\left[\alpha \right]}_{\text{D}}^{20}$ -107.8 (c 0.05, pyridine); ^1^H-NMR and ^13^C-NMR data, see Tables 3 and 4; HRESIMS (negative) *m*/*z*: 1223.5739 [M−H]^−^ (calc. for C_57_H_91_O_28_, 1223.5691).

Typaspidoside J (**9**): White amorphous power; ${\left[\alpha \right]}_{\text{D}}^{20}$ -41.9 (c 0.05, pyridine); ^1^H-NMR and ^13^C-NMR data, see Tables 3 and 4; HRESIMS (negative) *m*/*z*: 1223.5660 [M−H]^−^ (calc. for C_57_H_91_O_28_, 1223.5691).

Typaspidoside K (**10**): White amorphous power; ${\left[\alpha \right]}_{\text{D}}^{20}$ -55.1 (c 0.05, pyridine); ^1^H-NMR and ^13^C-NMR data, see Tables 3 and 4; HRESIMS (negative) *m*/*z*: 1223.5682 [M−H]^−^ (calc. for C_57_H_91_O_28_, 1223.5691).

Typaspidoside L (**11**): White amorphous power; ${\left[\alpha \right]}_{\text{D}}^{20}$ -56.4 (c 0.06, pyridine); ^1^H-NMR and ^13^C-NMR data, see Tables 3 and 4; HRESIMS (negative) *m*/*z*: 1193.5667 [M−H]^−^ (calc. for C_56_H_89_O_27_, 1193.5591).

Typaspidoside M (**12**): White amorphous power; ${\left[\alpha \right]}_{\text{D}}^{20}$ -80.6 (c 0.06, pyridine); ^1^H-NMR and ^13^C-NMR data, see Tables 3 and 4; HRESIMS (negative) *m*/*z*: 1209.5549 [M−H]^−^ (calc. for C_56_H_89_O_28_, 1209.5540).

### Acid hydrolysis of compounds 1–12

Compounds **1–12** (2.0 mg each) were treated in 2 N CF_3_COOH-H_2_O (2 mL) at 95°C for 4 h. The reaction mixture was extracted with CH_2_Cl_2_ (2 ml) three times. The aqueous layer was repeatedly evaporated to dryness until neutral. Then, in the monosaccharide mixture, glucose and galactose were detected by TLC analysis on a cellulose plate using n-BuOH-EtOAc-C_5_H_5_N-H_2_O (6:1:5:4) as the developing solution and aniline-o-phthalic acid as the detection solution and were then compared with the control samples: xyloses (R_f_ 0.56), glucose (R_f_ 0.45) and galactose (R_f_ 0.48). The sugar residue in pyridine (1 mL) was added to L-cysteine methyl ester hydrochloride (3.0 mg), and the mixture was kept at 60°C for 1 h. Then, HMDS-TMCS (hexamethyldisilazane- trimethylchlorosilane) (0.6 mL) was added to the reaction mixture, which was then kept at 60°C for 0.5 h. The supernatant (1.0 mL) was analyzed by GC under the following conditions: Agilent Technologies 6890 gas chromatograph was the equipment, carrying a 5973 mass spectrograph detector, and a HP-5 capillary column (30 m × 0.25 mm × 0.25 μm) was used. The conditions were as follow: column temperature: 180°C/250°C; programmed increase, 15°C/min; carrier gas: N_2_ (1 mL/min); injection and detector temperature: 250°C; injection volume: 4.0 μL; and split ratio: 1/50. The absolute configurations of the monosaccharides were confirmed by comparison of the retention times of the monosaccharide derivatives with those of standard samples: D-glucose (17.95 min) xylose (12.97 min) and D-galactose (18.57 min).

### HIV-1 inhibition assay of compounds 1–17

293T cells (2×10^5^) were co-transfected with 0.4 μg of pHIT/G and 0.6 μg of pNL-Luc-E. After 48h, the VSV-G pseudotyped viral supernatant (HIV-1) was harvested by filtration through a 0.45 μm filter and the concentration of viral capsid protein was determined by p24 antigen capture ELISA (Biomerieux). SupT1 cells were exposed to VSV-G pseudotyped HIV-1 (MOI = 1) at 37.0°C for 48 h in the absence or presence of the test compounds (Efavirenz was used as positive control). Viral infectivity was determined by measurement of luciferase activity in the infected cell using a Luciferase Assay (Promega), which was used to calculate the inhibition rate [14].

## Results and Discussion

**Fig 2:**
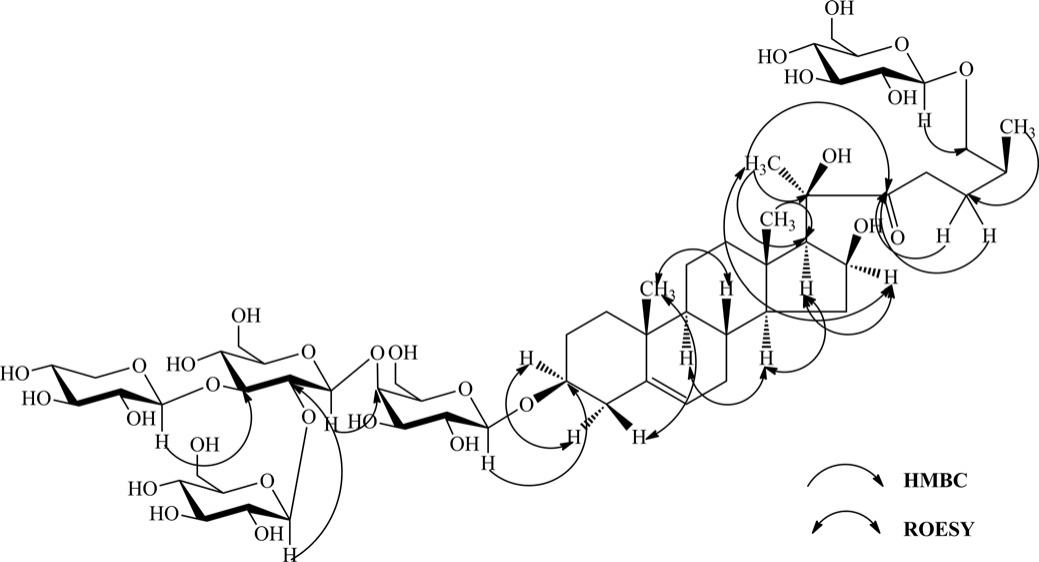
Key HMBC and NOE correlations of compound 1.

Compound **1** was isolated as a white amorphous powder. The negative high resolution electronic spray ion mass spectrometry (HRESIMS) showed an [M–H]^−^ion peak at *m/z* 1227.5665, corresponding to the molecular formula C_56_H_92_O_29_. The negative ESIMS showed fragment ions at *m/z* 1095.5, 1065.5, 915.5, 753.4, 591.3 and 429.3, attributable to the sequential losses of one pentose and four hexose residues and one molecule of water. The ^1^H NMR spectrum of **1** showed three methyl singlet signals at *δ* 1.38 (s, CH_3_-18), 0.87 (s, CH_3_-19) and 1.72 (s, CH_3_-21), one methyl doublet signal at *δ* 1.00 (d, *J* = 6.6 Hz, CH_3_-27), two methine protons indicative of secondary alcohols at *δ* 3.86 (m, H-3) and 4.83 (o, H-16), and one olefinic proton at *δ* 5.28 (br s, H-6). The ^13^C NMR spectrum of **1** showed 56 carbons, including two olefinic carbon signals at *δ* 141.1 (C-5) and 121.6 (C-6) and one ketone group signal at *δ* 215.8 (C-22). The presence of one ketone group located at C-22 and one hydroxyl group located at C-20 were supported by the fact that the HMBC correlations between protons H_2_-23 (*δ* 3.13), H_2_-24 (*δ* 1.60 and 2.13) and H_3_-21 (*δ* 1.72) and the carbon C-22 (*δ* 215.8), between the proton H_3_-21 (*δ* 1.72) and carbons C-20 (*δ* 82.5) and C-17 (*δ* 59.9).The *β* orientation of the oxygen atom at C-3, C-16 and C-20 positions was confirmed by the NOE enhancements of H-3 (*δ* 3.86)/Ha-1 (*δ* 0.94)/Hb-4 (*δ* 2.63), H-16 (*δ* 3.86) /H-17 (*δ* 1.86)/H-21 (*δ* 1.72), and Hb-1 (*δ* 1.64)/Ha-4 (*δ* 2.39)/H-19 (*δ* 0.87), while H-17 did not possess a NOE correlation with H-18 (*δ* 1.38). NOE correlations were also observed between H_3_-19/H-11, H-8/H_3_-18 and H-9/H-14 suggesting the usual trans junction for the B/C and C/D rings (Fig 2). The C-25 configuration was deduced to be (*S*) based on the difference of the chemical shifts of the geminal protons H_2_-26 (Δab = *δ* H_26a_ –*δ* H _26b_ = 0.57 ppm) [15]. Thus, the aglycone moiety of **1** was deduced as (20*R*,25S)-5-en-3*β*,16*β*,20,26-tetrol-16,22-secfurost-22-one, a new furostanol sapogenin with the cleavage of the E ring. The ^1^H and ^13^C NMR assignments of **1** (Tables 1 and 3) were established from the analysis of the ^1^H–^1^H COSY, HSQC and HMBC experiments. The anomeric regions in the ^1^H and ^13^C NMR spectra of **1** showed five anomeric proton signals at *δ* 4.86 (d, *J* = 7.8 Hz), 5.18 (d, *J* = 8.4 Hz), 5.57 (d, *J* = 7.8 Hz), 5.23 (d, *J* = 7.8 Hz) and 4.80 (d, *J* = 7.8 Hz) corresponding to the anomeric carbon signals at *δ* 102.8, 105.2, 104.9, 105.0 and 105.2. Glucose, galactose, and xylose were detected after acidic hydrolysis of **1** and their absolute configurations were identified as all D by GC analysis. The large coupling constants (^3^J_1,2_ > 7 Hz) were consistent with the *β* configuration of the sugar [16]. The long-range correlations between H-1'-Gal (*δ* 4.86) and C-3 (*δ* 78.2), H-1''-Glc (*δ* 5.18) and C-3'-Gal (*δ* 75.6), H-1'''-Glc (*δ* 5.57) and C-2''-Glc (*δ* 81.4), H-1''''-Xyl (*δ* 5.18) and C-3''-Glc (*δ* 86.8), and H-1'''''-Glc (*δ* 5.18) and C-26 (*δ* 75.6) in the HMBC spectrum revealed the sequence of the sugars and their linkage sites (Fig 2). Thus, the structure of **1** was elucidated as (20*R*,25*S*)-26-O-*β*-D- glucopyranosyl-5-en-3*β*,16*β*,20,26-tetrol-16,22-secfurost-22-one-3-*O*-*β*-D-glucopyranosyl-(1→2)-[*β*-D-glucopyranosyl]-(1→3)]*-β*-D-glucopyranosyl-(1→3)*-β*-D-galactopyranoside, named typaspidoside B.

Compound **2**, a white amorphous powder, showed an [M–H]^−^ion peak at *m/z* 1227.5659 in the negative HRESIMS, corresponding to the molecular formula C_56_H_92_O_29_. The negative ESIMS showed fragment ions at *m/z* 1095.5, 1065.5, 933.5, 771.4, 657.3 and 459.3, attributable to the sequential losses of one pentose and three hexose residues and one molecule of C_6_H_10_O_2_. A detailed comparison of the NMR data of **2** with those of **1** revealed that they shared similar aglycone and sugar moieties, except at the E ring of aglycone. The fact that C-22 position of the aglycone of **2** is one carboxyl group instead of the ketone group was deduced from the chemical shift of its C15~C18 and C19~C24 (*δ* 36.0, 75.4, 62.4, 13.3, 65.2, 24.2, 173.1, 32.5 and 29.2 instead of *δ* 37.5, 72.9, 59.9, 15.8, 82.5, 28.6, 215.8, 36.0 and 27.7 in compound **1**) (Table 1). In the HMBC spectrum, the long-range correlations of H-21 (*δ* 1.43) with C-20 (*δ* 65.2) and C-17 (*δ* 62.4), and H_2_-24 (*δ* 1.58 and 1.99) with C-22 (*δ* 173.1) and C-27 (*δ* 16.9) revealed the ring crack was between C-20 and C-22 and the carboxyl group was substituted at C-22 of the aglycone. The NOE correlations between H-21 (*δ* 1.43) and H-17 (*δ* 1.58) and H-16 (*δ* 5.33) revealed the *β* orientation of the oxygen atom at C-16 and C-20. Thus, the aglycone moiety of **2** was deduced as (20*S*,25*S*)-5-en-3*β*,20*β*,26-triol-20,22-secfurost-22-one, an unreported sapogenin. The sugar unit and its linkage site to the aglycone were identified as in **1**. Thus, **2** was determined to be (20*S*,25*S*)-26-O-*β*-D-glucopyranosyl-5-en-3*β*,20*β*,26-triol-20,22-secfurost-22-one-3-*O*-*β*-D-glucopyranosyl-(1→2)-[*β*-D-glucopyranosyl]-(1→3)]*-β*-D-glucopyranosyl-(1→3)*-β*-D-galactopyranoside, named typaspidoside C.

Compound **3** was isolated as a white amorphous powder with a molecular formula of C_56_H_88_O_30_, as determined by HRESIMS (*m/z*: 1239.5238 [M−H]^−^). The negative ESIMS showed fragment ions at *m/z* 945.4, 813.3, 783.3, 651.3, 489.2 and 327.2. A detailed comparison of the NMR data of **3** with those of **2** revealed that they shared similar sugar moieties, but significant differences in the chemical shifts of B~E rings of the aglycone. The ^1^H NMR spectrum of **3** showed three methyl singlet signals at *δ* 1.52 (s, CH_3_-18), 0.90 (s, CH_3_-19) and 2.26 (s, CH_3_-21), two methine protons at *δ* 3.27 (d, *J* = 7.8 Hz, H-20) and 5.62 (o, H-16). The ^13^C NMR spectrum of **3** showed two olefinic carbon signals at *δ* 141.0 (C-5) and 121.2 (C-6), one carboxyl group signal at *δ* 173.3 (C-22) and two ketone group signals at *δ* 211.9 (C-12) and 204.4 (C-20). The fact that two ketone groups were located at C-12 and C-20 were supported the long-range correlations of H_2_-11 (*δ* 2.57 and 2.23), H_2_-41 (*δ* 1.18), H-17 (*δ* 3.27) and H_3_-18 (*δ* 1.52) with C-12 (*δ* 211.9), and H-17 (*δ* 3.27) and H_3_-21 (*δ* 2.26) with C-20 (*δ* 204.4) in the HMBC spectrum. Thus, the aglycone moiety of **3** was deduced as (25*S*)-5-en-3*β*,26-diol-20,22-secfurost-12,20,22-trione [15,17], an unreported sapogenin with the E ring cleavage. Through analysis of the 1D- and 2D-NMR data of **3**, its structure was determined as (25*S*)-26-O-*β*-D-glucopyranosyl-5-en-3*β*,26-diol-20,22-secfurost- 12,20,22-trione-3-*O*-*β*-D-glucopyranosyl-(1→2)-[*β*-D-xylopyranosyl-(1→3)]-*β*-D-glucopyranosyl-(1→4)-*β*-D-galactopyranoside, named typaspidoside D.

Compound **4** displayed an [M−H]^**−**^ ion peak at *m*/*z* 1225.5524 in the negative HRESIMS, indicating a molecular formula of C_56_H_90_O_29_. A detailed comparison of the MS and NMR data of **4** with those of **3** revealed that they shared similar structures but exhibited significant differences in the B ring of the aglycone. The lack of one ketone group at position C-12 in **4** was deduced from the chemical shift of its C-9~C-13 and C-17 (Table 1). The sugar units and their linkage sites were identified as in **1~3.** Thus, the structure of **4** was established as (25*S*)-26-O-*β*-D-glucopyranosyl- 5-en-3*β*,26-diol-20,22-secfurost-20,22-dione-3-*O*-*β*-D-glucopyranosyl-(1→2)-[*β*-D-xylopyranosyl-(1→3)]-*β*-D-glucopyranosyl-(1→4)-*β*-D-galactopyranoside, named typaspidoside E.

Compound **5** was isolated as a white amorphous solid. Its molecular formula C_56_H_92_O_29_ was deduced from the pseudo-molecular ion peak at *m/z* 1227.5681 [M–H]^−^in the HRESIMS. The existence of four hexose units and one pentose unit was proved by the fragment ions at *m/z* 1095.5, 1065.5, 933.5, 771.4, 609.4 and 429.3 in the negative ESI-MS. The ^1^H NMR spectrum showed two singlet signals at *δ* 1.14 (3H, s, CH_3_-18) and 0.90 (3H, s, CH_3_-19), two doublet signals at *δ* 1.59 (3H, d, *J* = 6.6 Hz, CH_3_-21) and 1.01 (3H, d, *J* = 7.2 Hz, CH_3_-27), and three oxygenated methine protons at *δ* 3.84 (m, H-3), 3.57 (m, H-12) and 5.02 (m, H-16). A detailed comparison of the NMR data of **5** with those of **4** revealed that they shared similar sugar moieties, except that the C~E rings of the aglycone lack three carbonyl groups. The long range correlations of C-12 (*δ* 79.1) with H_2_-11 (*δ* 1.67 and 1.85), H-14 (1.11), H-17 (2.30) and H-18 (1.14) in the HMBC spectrum and the cross-peak correlations between H-12 (*δ* 3.57) and H_2_-11 (*δ* 1.67 and 1.85) in the COSY spectrum indicated that the hydroxyl group was located at the C-12 of the aglycone. The *β* orientation of OH-12 was confirmed by the NOE correlations between H-12/H-9/H-14/H-11/H-17 in the ROESY spectrum [18,19]. The *α* orientation of the hydroxyl group at C-22 was established by the semiketal carbon signal at *δ* 110.9 [20,21]. Therefore, the structure of **5** was assigned as 26-*O*-*β*-D-glucopyranosyl-(25*S*)-furost-5-en-3*β*,12*β*,22*α*,26-tetrol-3-*O*-*β*-D-glucopyranosyl-(1→2)-[*β*-D-xylopyranosyl-(1→3)]-*β*-D-glucopyranosyl-(1→4)-*β*-D-galactopyranoside, named typaspidoside F.

Compound **6** showed an [M−H]^−^ ion peak at *m/z* 1225.5472 in the negative HRESIMS, indicating a molecular formula of C_56_H_90_O_29_. In the ^13^C NMR spectrum, the carbon signals at *δ* 165.3 (C-5), 126.1 (C-6) and 201.0 (C-7) indicated the aglycone possess a conjugate ketone and double bond. In the HMBC spectrum, H_2_-4 (*δ* 2.44 and 2.73) and H-19 (0.95) showed long range correlations to C-5 (*δ* 165.3), and H-8 (*δ* 2.38) and H-14 (*δ* 1.48) showed long range correlations to C-7 (*δ* 201.0) indicated that the *α*,*β*-unsaturated carbonyl structure was located at the C-5~C-7 positions of the aglycone [22]. Comparison of the NMR data of **6** with those of **5**, the structure of **6** was elucidated as 26-*O*-*β*-D-glucopyranosyl-(25*S*)-furost-5-en-3*β*,22α,26-triol-7- one-3-*O*-*β*-D-glucopyranosyl-(1→2)-[*β*-D-xylopyranosyl-(1→3)]-*β*-D-glucopyranosyl-(1→4)-*β*-D-galactopyranoside, named typaspidoside G.

Compound **7** was isolated as a white amorphous powder. The negative HRESIMS showed an [M−H]^−^ ion peak at *m*/*z* 1209.5521, corresponding to a molecular formula of C_56_H_90_O_28_. The ^1^H NMR spectrum of **7** showed three methyl singlet signals at *δ* 0.89 (s, CH_3_-18), 0.89 (s, CH_3_-19) and 1.71 (s, CH_3_-21), one methyl doublet signal at *δ* 1.07 (d, *J* = 6.6 Hz, CH_3_-27). Comparing the MS and NMR data with those of **5**, compound **7** was determined to contain the similar sugar moieties, but significant differences in the chemical shifts of the aglycone. Through analysis of the 1D- and 2D-NMR data of **7** (Table 2), the lack of two hydroxyl groups at position C-12 and C-22, but possess a hydroxyl group at position C-20 and one doubled bond at C-22 and C-23 of the aglycone in **7** could be deduced. This was confirmed by the HMBC correlations of H_3_-21 (*δ* 1.71) with C-17 (*δ* 67.9), C-20 (*δ* 76.7) with C-22 (*δ* 163.8), C-23 (*δ* 91.4) and H_2_-24 (*δ* 2.13 and 2.51) with H-25 (*δ* 2.09), and H-17 (*δ* 2.22) with C-12 (*δ* 39.3), C-13 (*δ* 40.4), C-20 (*δ* 76.7) and C-22 (*δ* 163.8). NOE correlations between H-21 and H-18 were indicative of *α* orientation of OH-20. The aglycone is same as the aglycone of compound 1 in the literature [23]. Thus, the structure of **7** was identified as 26-*O*-*β*-D-glucopyranosyl-(20*S*,25*S*)-furost-5,22(23)-dien-3*β*,16*β*,20,26-tetrol-3-*O*- *β*-D-glucopyranosyl-(1→2)-[*β*-D-xylopyranosyl-(1→3)]-*β*-D-glucopyranosyl-(1→4)-*β*-D-galactopyranoside, named typaspidoside H.

Compound **8** showed an [M−H]^**−**^ ion peak at *m*/*z* 1223.5739 in the negative HRESIMS, corresponding to a molecular formula of C_57_H_92_O_28_. A detailed comparison of the MS and NMR data of **8** with those of **7** revealed that they shared similar aglycone and sugar moieties, except at the E ring of the aglycone possess an additional methoxy at position C-20. This was confirmed by the HMBC correlation between H_3_-21 (*δ* 1.37) and C-17 (*δ* 66.7), C-20 (*δ* 82.4) and C-22 (*δ* 157.3), and OCH_3_-20 (*δ* 3.17) and C-20 (*δ* 82.4). NOE correlations of H-17/H-21/H-16 were indicative of *β* orientation of OCH_3_-20 [23]. Thus, the structure of **8** was elucidated as 26-*O*-*β*-D-glucopyranosyl-(20*R*,25*S*)-furost-5,22(23)-dien-20*β*-methoxy-3*β*,16*β*,26-triol-3-*O*-*β*-D-glucopyranosyl-(1→2)-[*β*-D-xylopyranosyl-(1→3)]-*β*-D-glucopyranosyl-(1→4)-*β*-D-galactopyranoside, named typaspidoside I.

Compounds **9** and **10** showed the [M−H]^**−**^ ion peaks at *m*/*z* 1223.5660 and 1223.5682 in the negative HRESIMS, indicating the same molecular formula of C_57_H_92_O_28_. A detailed comparison of the MS and NMR data of **9** and **10** with those of **8** revealed that they shared similar aglycone and sugar moieties, except at the E and F rings of the aglycone. In the HMBC spectrum, long rang correlations were observed between H_3_-21 (*δ* 1.73) and C-17 (*δ* 64.8), C-20 (*δ* 109.0) and C-22 (*δ* 15.7), and between H_3_-OCH_3_ (*δ* 3.35) and C-23 (*δ* 73.1) confirming the OCH_3_ at position C-23 and a double bond at position C-20(22) of the aglycone. Comparing the NMR data with those of dioscoresides C and E [24,25], **9** was determined to contain the same aglycone as dioscoreside E, but difference chemical shifts of H_2_-26 (*δ* 3.53 and 4.06). So, the structure of **9** was elucidated as 26-*O*-*β*-D-glucopyranosyl-(23*S*,25*S*)-furost-5,20(22)-dien-23-methoxy-3*β*,16*β*,26-triol-3-*O*-*β*-D-glucopyranosyl-(1→2)-[*β*-D-xylopyranosyl-(1→3)]-*β*-D-glucopyranosyl-(1→4)-*β*-D-galactopyranoside, named typaspidoside J.

The different orientation of methoxy at C-23 in **10** was deduced from the chemical shift of its H-23 and H-24 (*δ* 4.22, 1.86 and 2.06 instead of *δ* 4.18, 1.63 and 2.29 in **9**). So, the structure of **10** was elucidated as 26-*O*-*β*-D-glucopyranosyl-(23*R*,25*S*)-furost-5,20(22)-dien-23-methoxy-3*β*,16*β*, 26-triol-3-*O*-*β*-D-glucopyranosyl-(1→2)-[*β*-D-xylopyranosyl-(1→3)]-*β*-D-glucopyranosyl-(1→4)-*β*-D-galactopyranoside, named typaspidoside K

Compound **11** displayed an [M−H]^**−**^ ion peak at *m*/*z* 1193.5667 in the negative HRESIMS, indicating a molecular formula of C_56_H_90_O_27_. A detailed comparison of the NMR and MS data of **11** with those of **10** revealed that they shared similar aglycone and sugar moieties, except the lack of one methoxy at position C-23 of the aglycone in **11**. So, the structure of **11** was defined as 26-*O*-*β*-D-glucopyranosyl-(25*S*)-furost-5,20(22)-dien-3*β*,16*β*,26-triol-3-*O*-*β*-D-glucopyranosyl-(1→2)-[*β*-D-xylopyranosyl-(1→3)]-*β*-D-glucopyranosyl-(1→4)-*β*-D-galactopyranoside, named typaspidoside L.

Compound **12** was isolated as a white amorphous powder. The molecular formula was determined as C_56_H_90_O_28_ by the [M−H]^−^ ion peak at *m*/*z* 1209.5549 in the negative HRESIMS. The ^1^H NMR spectrum of **12** showed four methyl signals at *δ* 0.74 (s, CH_3_-18), 0.86 (s, CH_3_-19), 1.09 (d, *J* = 7.2 Hz, CH_3_-21) and 1.31 (d, *J* = 6.6 Hz, CH_3_-27), and three methine proton signals of CHOR at *δ* 3.86 (m, H-3), 4.43 (o, H-16) and 4.80 (td, *J* = 5.4, 10.8 Hz, H-24). The hydroxyl group at the C-24 position was confirmed by the HMBC corrections of H_3_-27 (*δ* 1.31) with C-24 (*δ* 72.9), C-25 (*δ* 31.8) and C-26 (*δ* 64.2). A detailed comparison of the NMR and MS data of **12** with those of **10**, terrestrinin I [21] and 1b [26] revealed that **12** and **10** shared similar A-C rings and sugar moieties at C-3 position, and **12** and 1b shared similar D-F rings. The C-24*S* and C-25*R* configurations were also deduced from the *J* values of 10.8 Hz (H-24/H-23ax), 5.4 Hz (H-24/H-23 eq), 5.4 Hz (H-24/H-25), and br s (H-25/H-26 ax). Therefore, the aglycone of **12** was identified as (24*S*,25*R*)-5-en-spirostan-3*β*,24*β*-diol. The ^1^H and ^13^C NMR spectra showed anomeric proton at *δ* 5.02 (d, *J* = 8.4 Hz), corresponding to the anomeric carbon at *δ* 101.2. The HMBC correction between H-1''''' (*δ* 5.02) and C-24 (72.9) revealed the sugar linkage site. Therefore, the structure of **12** was assigned as 24-*O*-*β*-D-glucopyranosyl-(24*S*,25*R*)-5-en-spirostan-3*β*,24*β*-diol-3-*O*-*β*-D-glucopyranosyl-(1→2)-[*β*-D-xylopyranosyl-(1→3)]-*β*-D-glucopyranosyl-(1→4)-*β*-D-galactopyranoside, named typaspidoside M.

**Fig 3:**
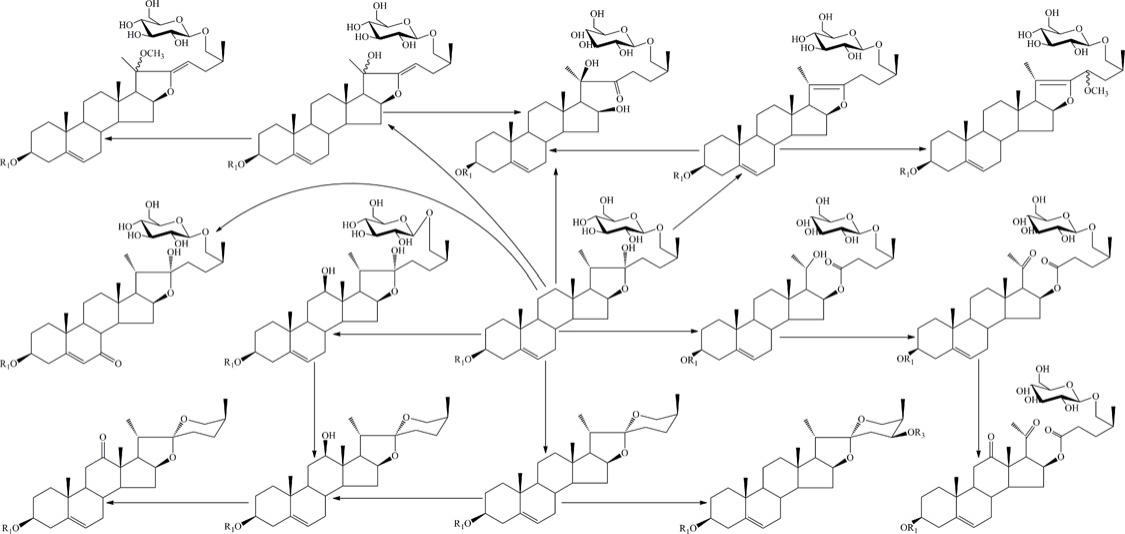
The plausible biosynthetic relationship between the isolated compounds.

There are sixteen aglycones in compounds **1**–**17**, typaspidoside A1 and timosaponin H_1_ [7], and they have different oxidative levels. Compounds **1~11**, typaspidoside A1 and timosaponin H_1_ are furostanol saponins and **12~17** are spirostanol saponins. Compounds **13**, **14** and **16** are the corresponding spirostanol type of **5**, typaspidoside A1 and timosaponin H_1_, respectively. The proto-yamogenin may be the common evolutionary precursor for all compounds in this paper. Compounds **1**, **2**, **5** and **6** are the secondary oxidation level products of timosaponin H_1_, and **11** is a dehydroxylation product of timosaponin H_1_. Compound **7** was formed through dehydroxylation and hydroxylation from timosaponin H_1_. Compound **8** was a methylate of **7.** Compounds **9** and **10** are hydroxylation and methylation products of **11**. Compound **3** is the tertiary oxidation level product of timosaponin H_1_. Compounds **12**, **13** and **14** are the secondary oxidation level products of **16**, furthermore, **12** is a two chains saponin after glycosidation. The detail plausible biosynthetic relationship between the isolated compounds was shown in Fig 3.

Compounds **1**–**17** were evaluated for anti-HIV activities using one-round infectivity assay and the results were shown in **Table 5**. All test compounds exhibited anti-HIV activities to different degrees, and 9 of them inhibited viral replication by more than 50% at a concentration of 30 uM. The spirostanol saponins showed the more strong activities than furostanol saponins. Especially, 4 compounds (**13**, **14**, **16** and **17**) showed potent activities against HIV-1with >95% inhibition of viral replication, which have the potential for anti-HIV drug development and are worth to exploit further.

**Table 5:** The anti-HIV activities of the isolated steroidal glycosides from the rhizomes of *A*. *typical*.

Compounds	Dose	Inhibition rate (%)	Compounds	Dose	Inhibition rate (%)
Efavirenz	1.5nM	0.99	**9**	30 μM	50.15±4.51
**1**	30 μM	47.72±2.74	**10**	30 μM	43.43±0.34
**2**	30 μM	48.42±1.87	**11**	30 μM	41.94±3.80
**3**	30 μM	29.42±5.61	**12**	30 μM	49.17±3.65
**4**	30 μM	51.99±0.77	**13**	30 μM	95.14±2.52
**5**	30 μM	46.20±7.23	**14**	30 μM	99.95±0.01
**6**	30 μM	80.78±0.07	**15**	30 μM	62.19±2.65
**7**	30 μM	51.00±0.07	**16**	30 μM	99.95±0.02
**8**	30 μM	48.63±2.38	**17**	30 μM	98.47±1.57

## Supporting Information

S1 File MS, 1D and 2D NMR spectra of compounds 1–12 are available as Figures Aa‒Ld. (DOC)
