# Supplementary material for: Steroidal Saponins from the Rhizomes of Aspidistra typica
Source: PLoS One. 2016 Mar 3;11(3):e0150595. doi: 10.1371/journal.pone.0150595 (PMC4777403; doi:10.1371/journal.pone.0150595)
Supplement: S1 File — (DOC) [file pone.0150595.s001.doc]

**Supporting Information**

Jiang-Ming Cui ^1,2¶^ Li-Ping Kang ^3¶^, Yang Zhao ^1^, Jian-Yuan Zhao ^4^, Jie Zhang ^5^, Xu Pang ^4^, He-Shui Yu ^1^, De-Xian Jia ^5^, Chao Liu ^1^, Li-Yan Yu ^4^, Bai-Ping Ma ^1*^

# Affiliation

^1^ *Beijing Institute of Radiation Medicine, Beijing 100850, PR China;*

*^2^ School of Pharmaceutical Sciences, Central South University, Changsha 410013, China*

*^3^* *State Key Laboratory Breeding Base of Dao-di Herbs, National Resource Center for Chinese Materia Medica, China Academy of Chinese Medical Sciences, Beijng, 100700, People’s Republic of China;*

*^4^ Institute of Medicinal Biotechnology, Chinese Academy of Medical Sciences & Peking Union Medical College, Beijing 100050 , China;*

*^5^* *Ovation Health Science and Technology Co.Ltd., ENN Group, Langfang 065001, China*

* Corresponding author

E-mail: mabaiping@sina.com (BPM)

^¶^ These authors contributed equally to this work.

Figure Aa HR-ESI-MS of compound **1**


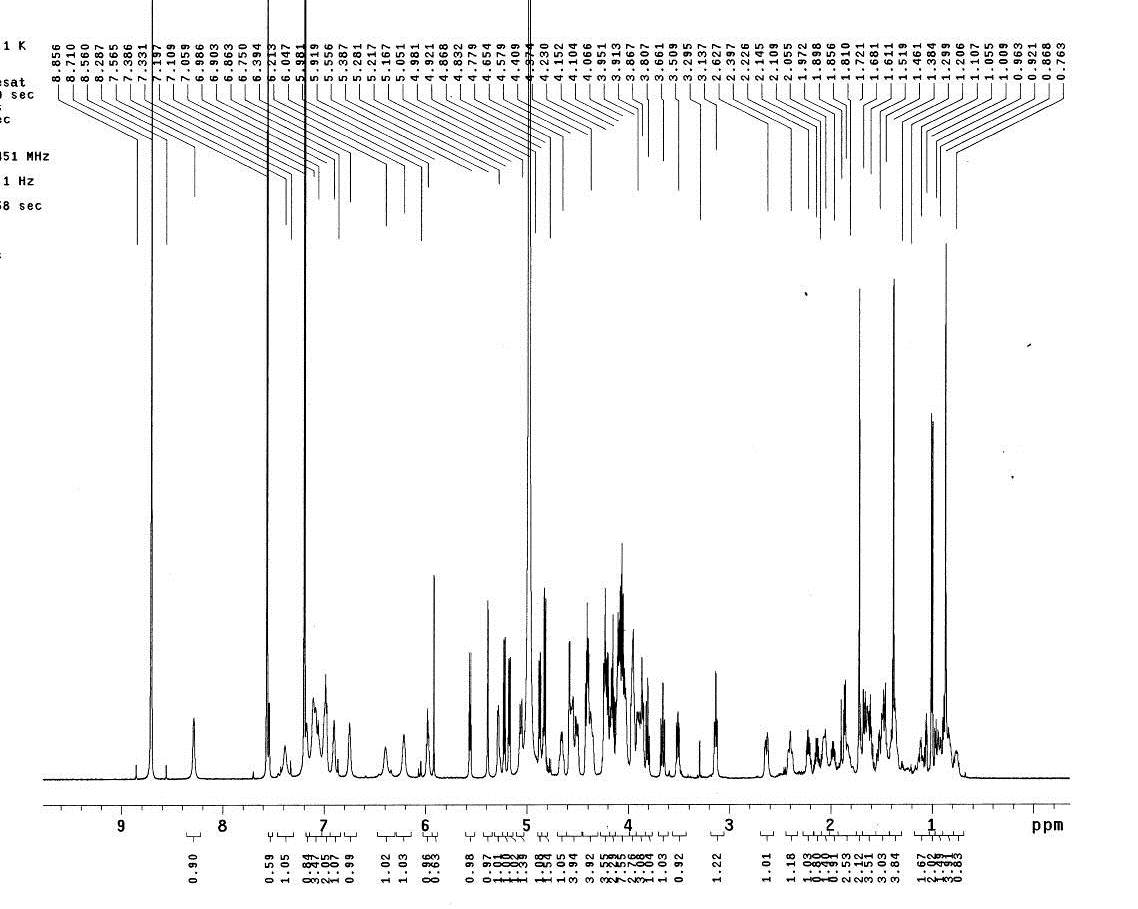


Figure Ab. ^1^H-NMR of compound **1**


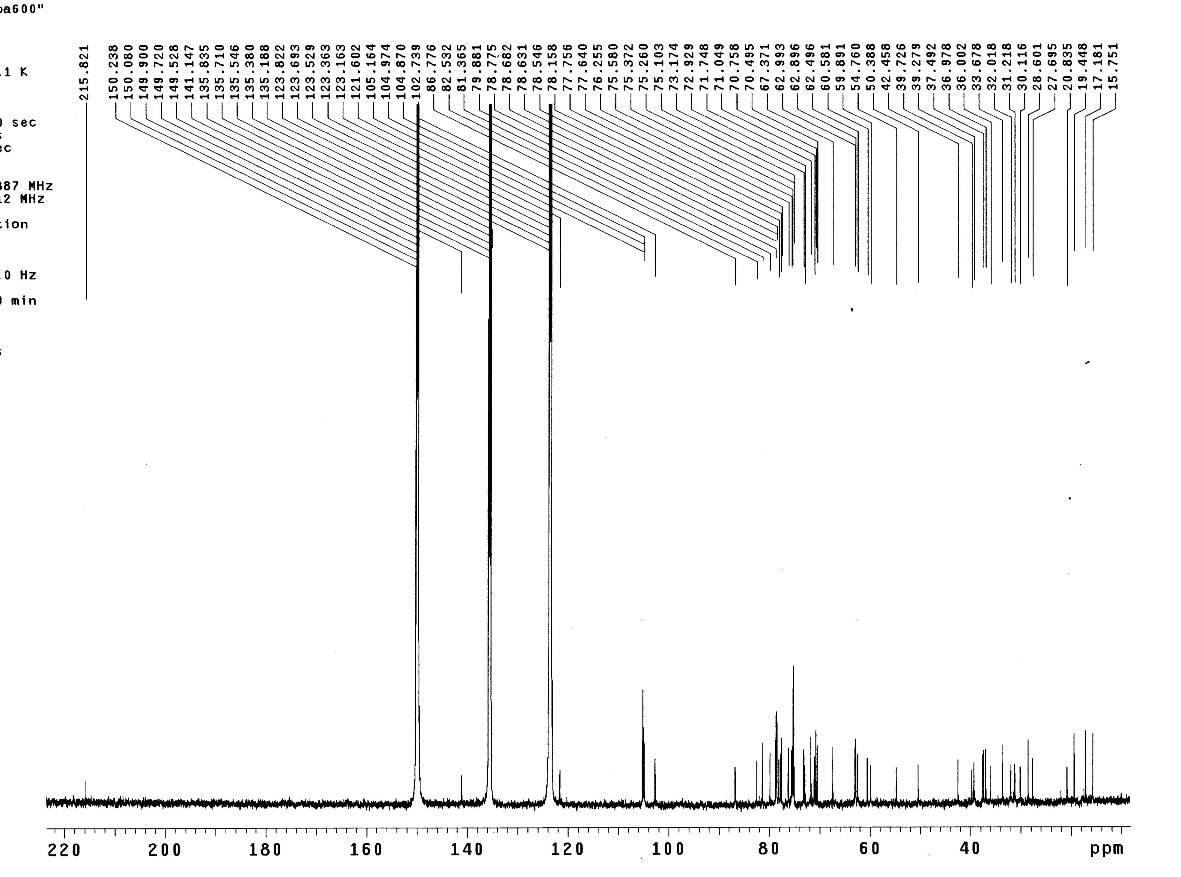


Figure Ac Fig. ^13^C-NMR of compound **1**


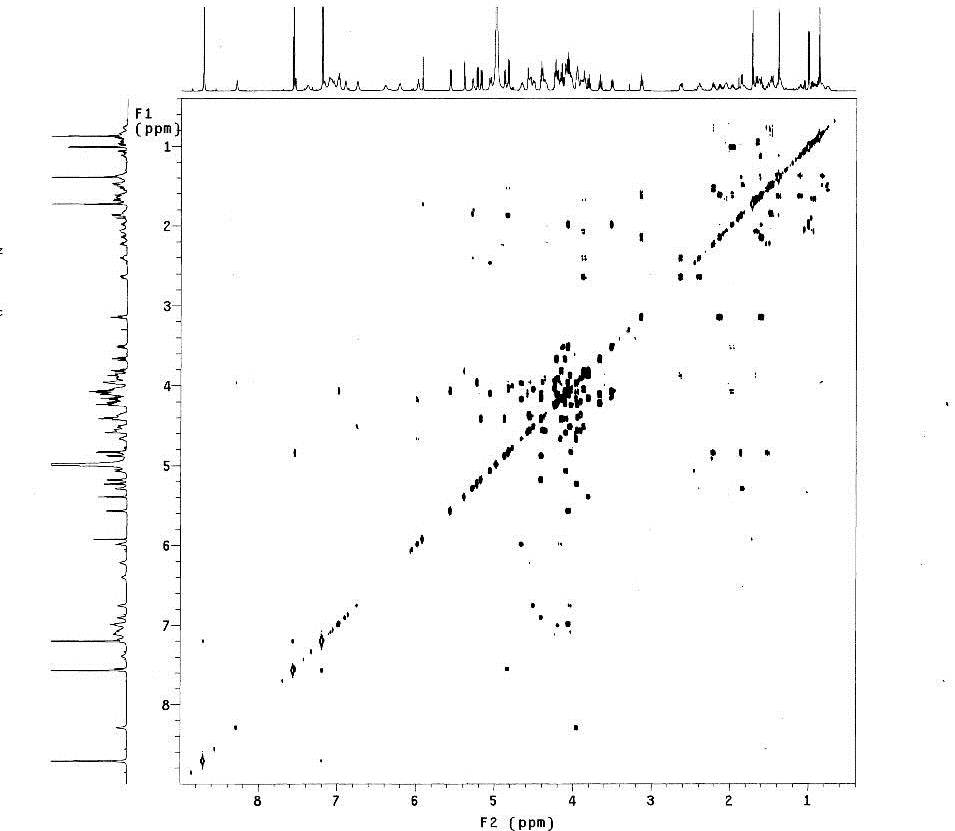


Figure Ad ^1^H-^1^H COSY of compound **1**


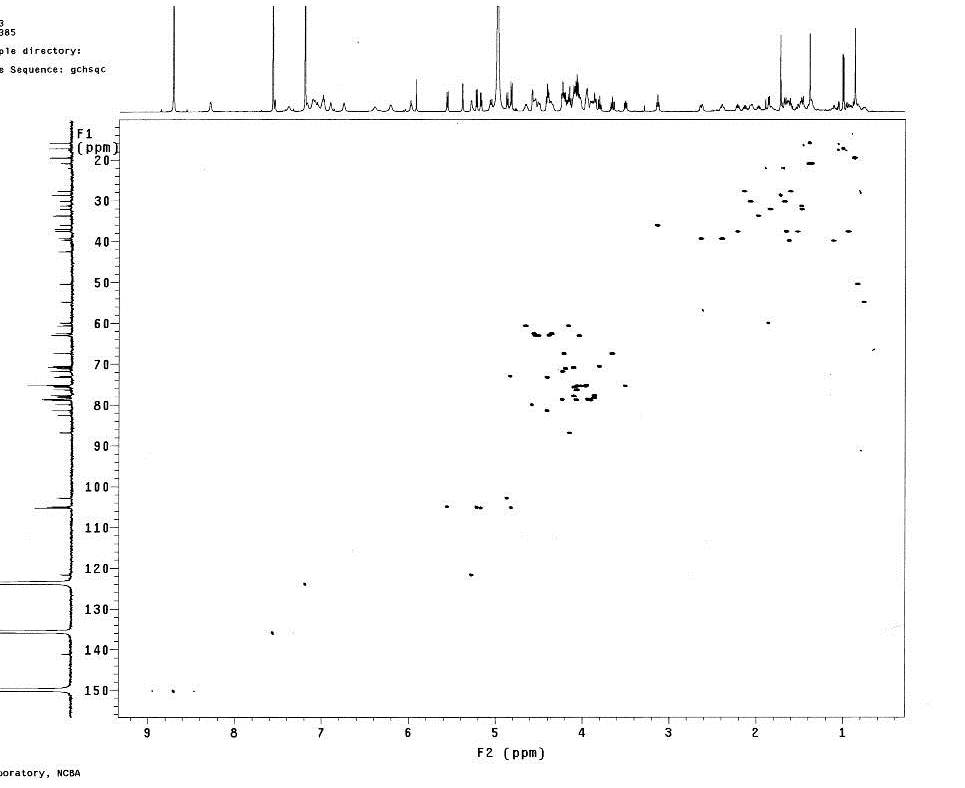


Figure Ae HSQC of compound **1**


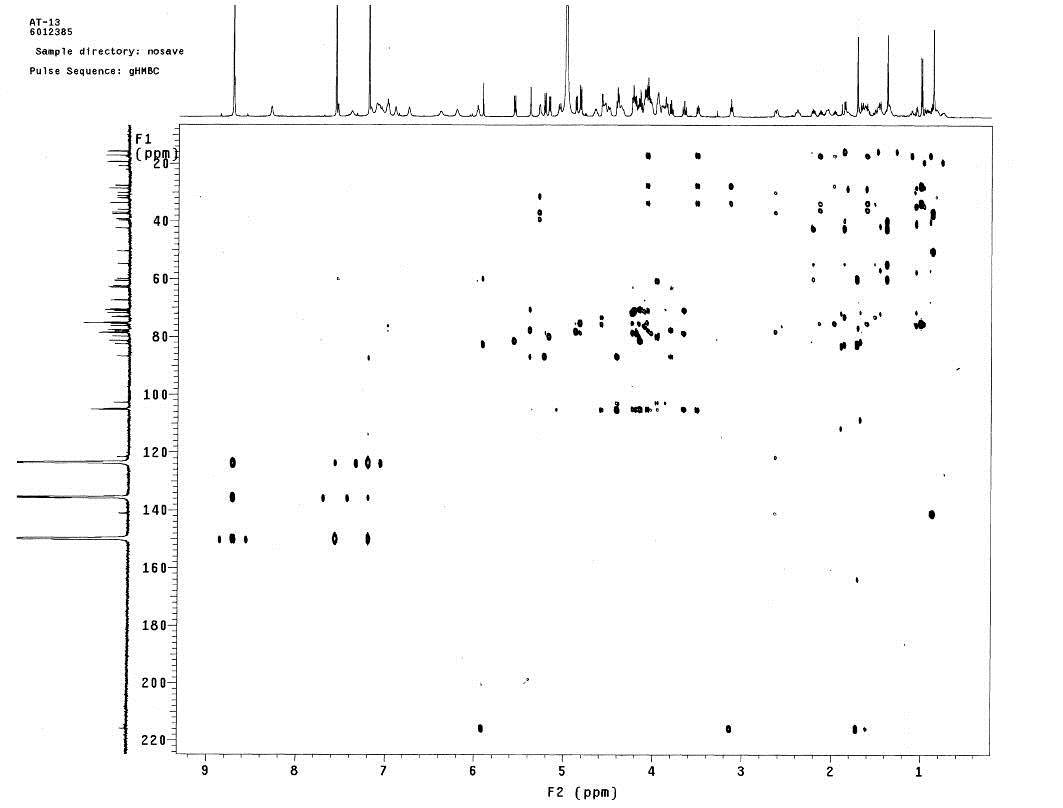


Figure Af HMBC of compound **1**


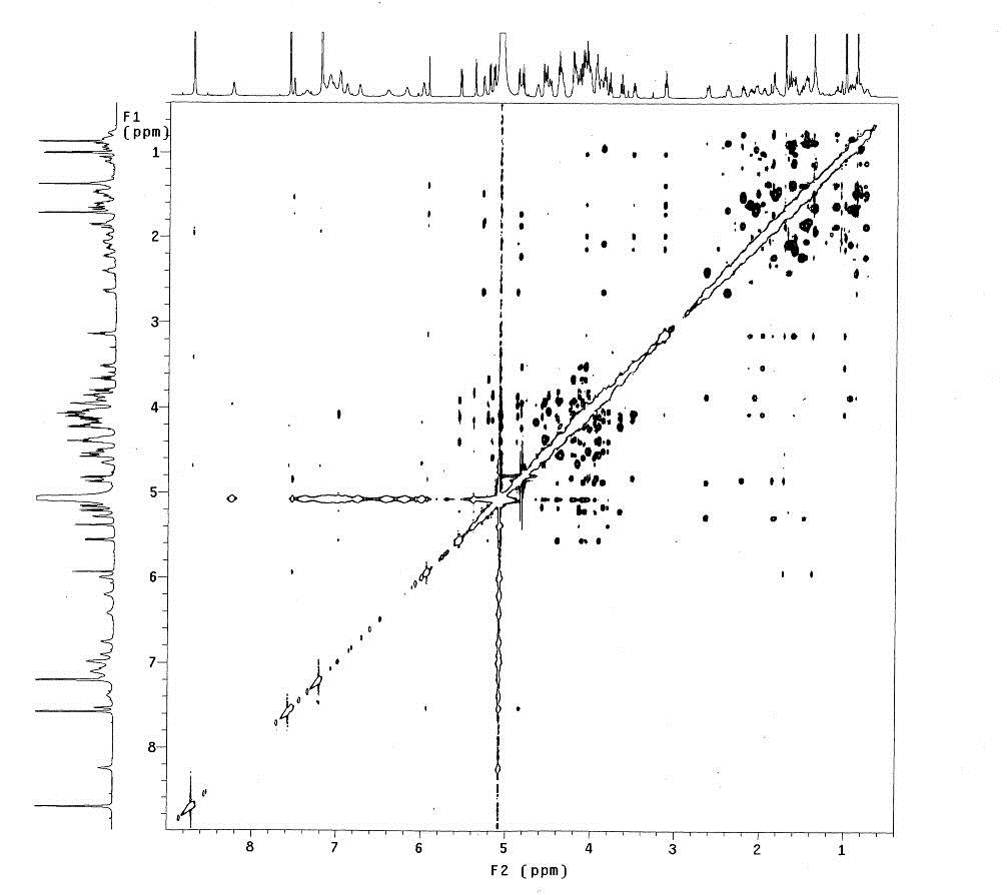


Figure Ag NOESY of compound **1**

Figure Ba HR-ESI-MS of compound **2**


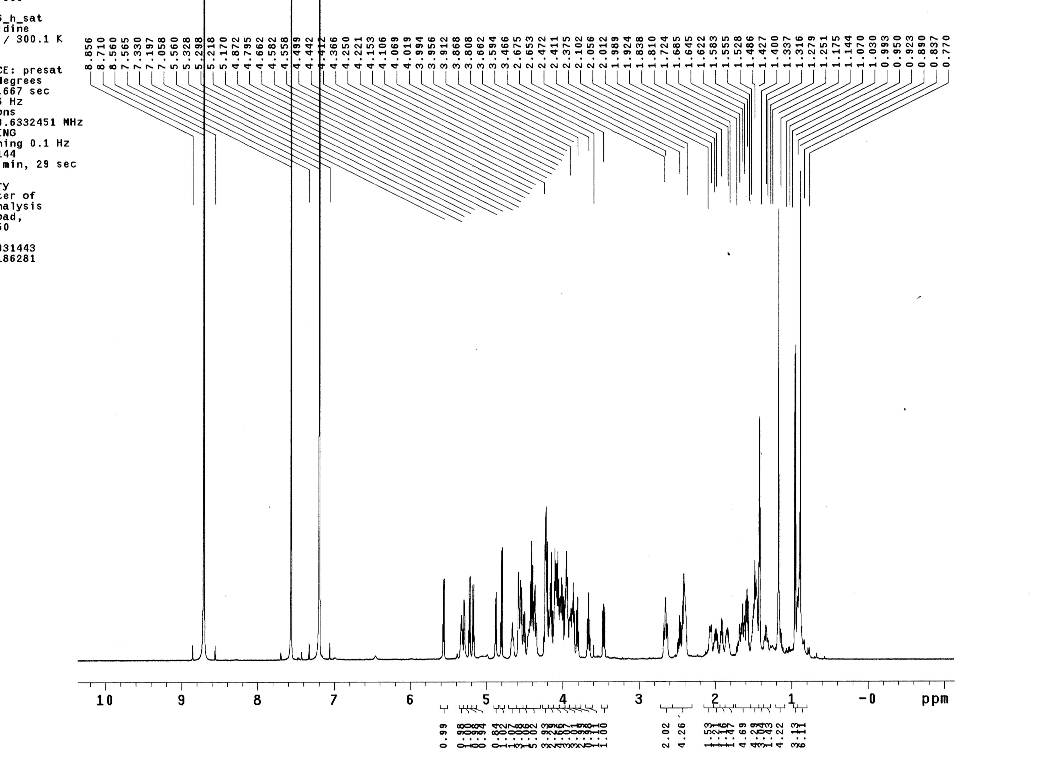


Figure Bb ^1^H-NMR of compound **2**

**
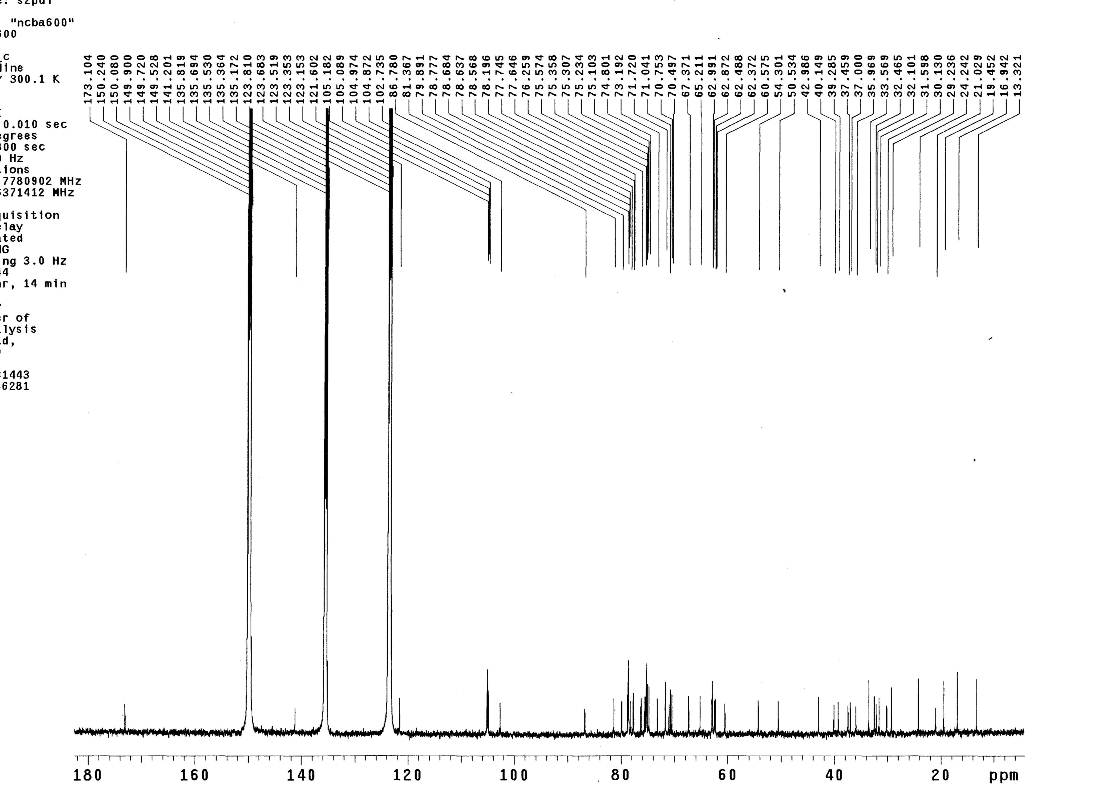
**

Figure Bc ^13^C-NMR of compound **2**


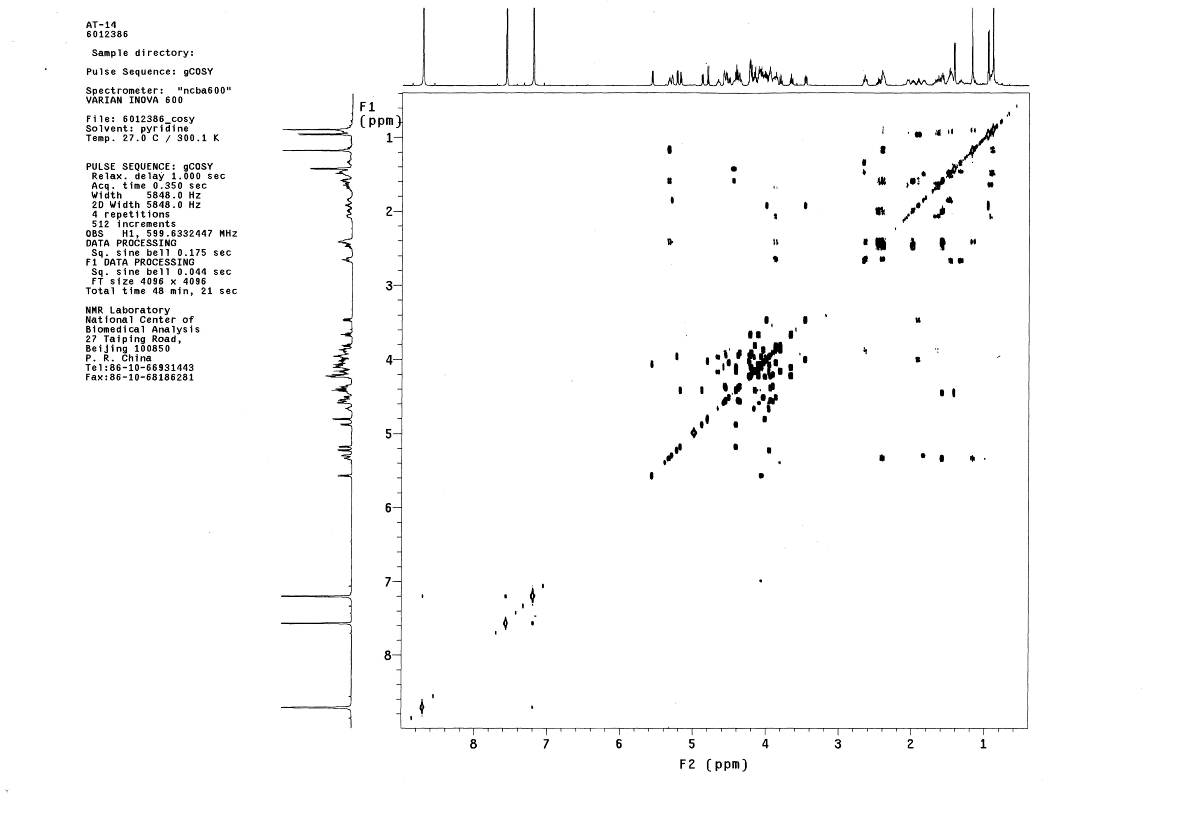


Figure Bd ^1^H-^1^H COSY of compound **2**

Figure Be HSQC of compound **2**


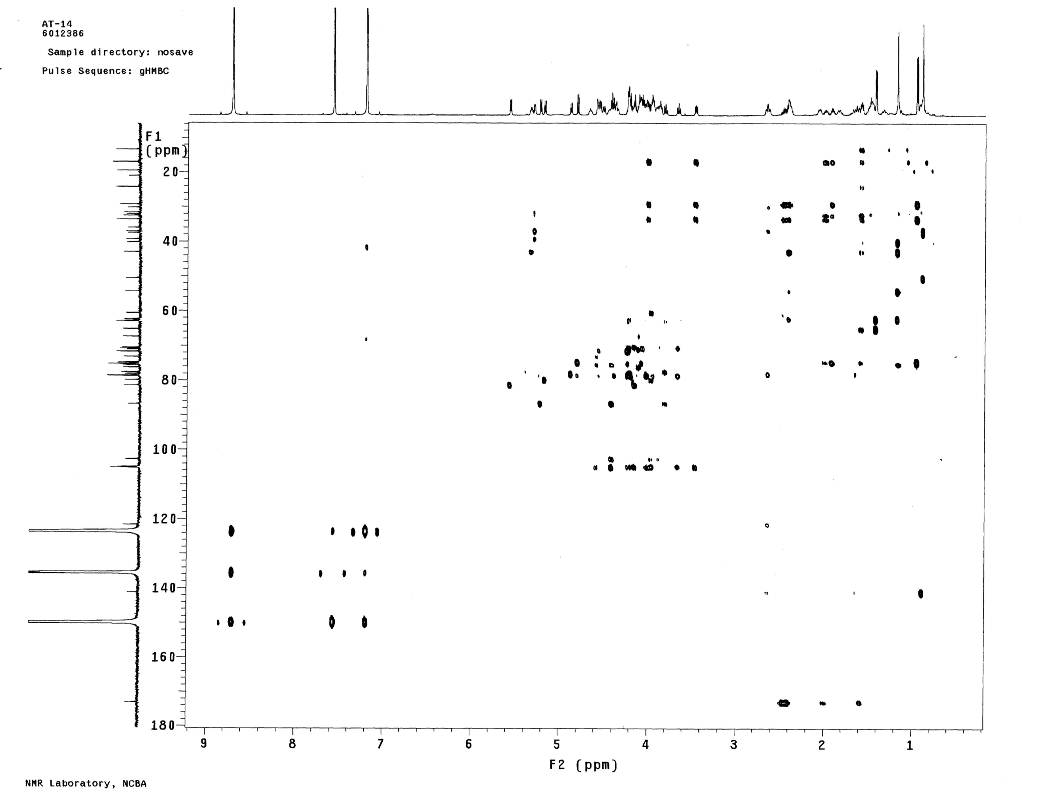


Figure Bf HMBC of compound **2**

Figure Ca HR-ESI-MS of compound **3**


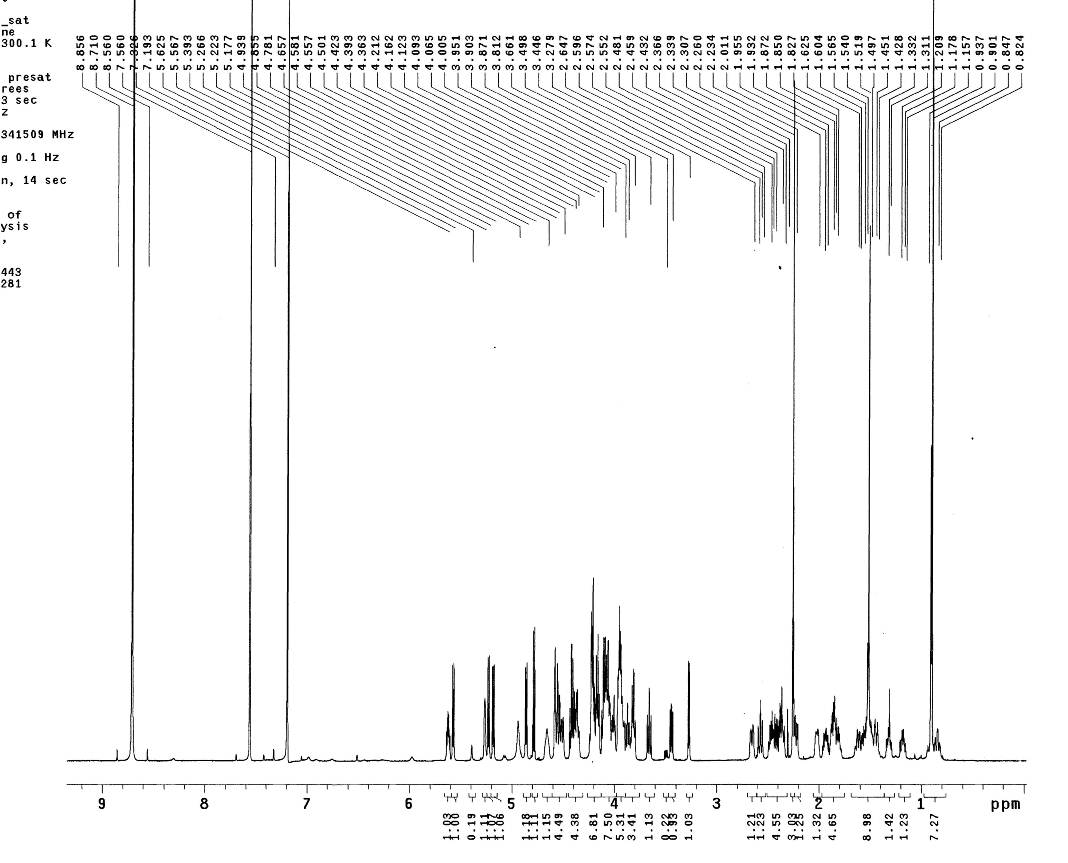


Figure Cb ^1^H-NMR of compound **3**


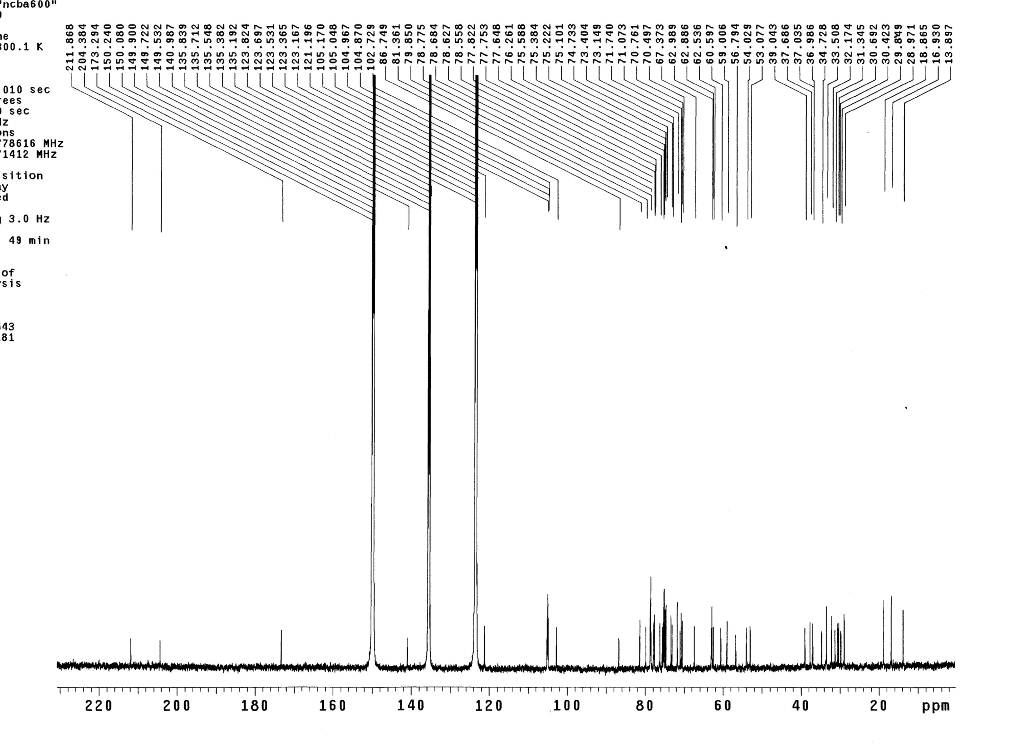


Figure Cc ^13^C-NMR of compound **3**


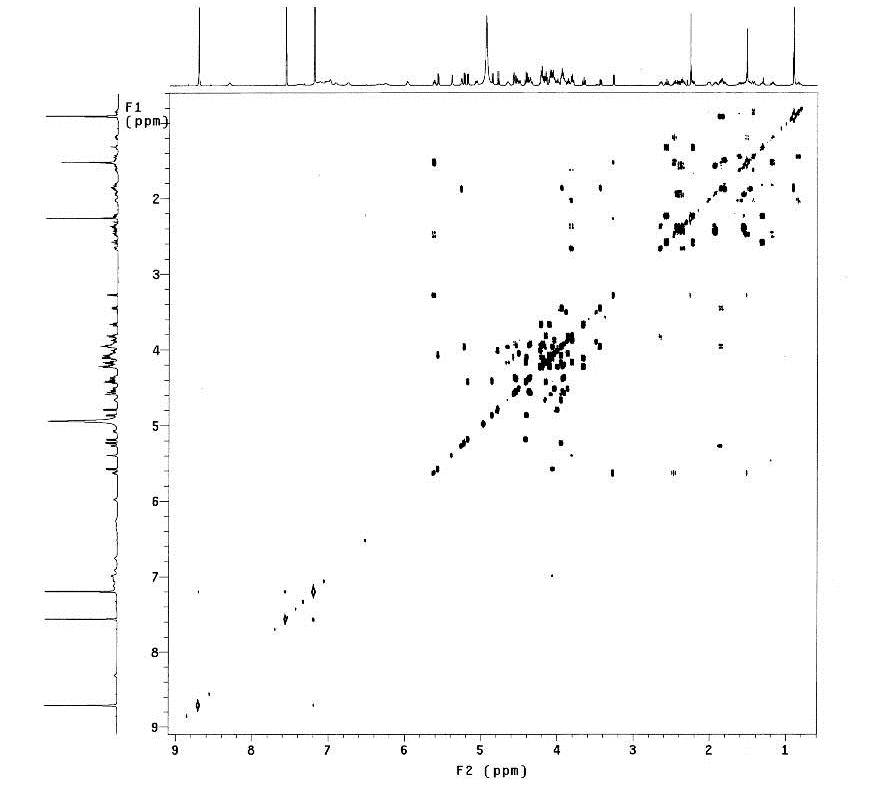


Figure Cd COSY of compound **3**

**
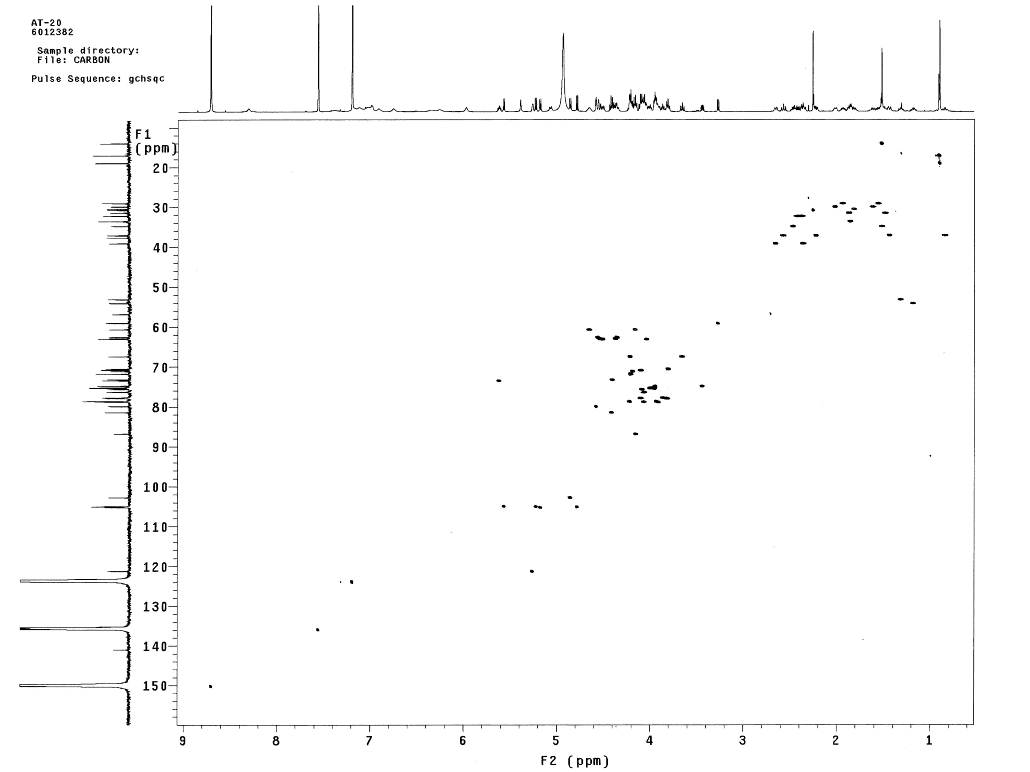
**

Figure Ce HSQC of compound **3**


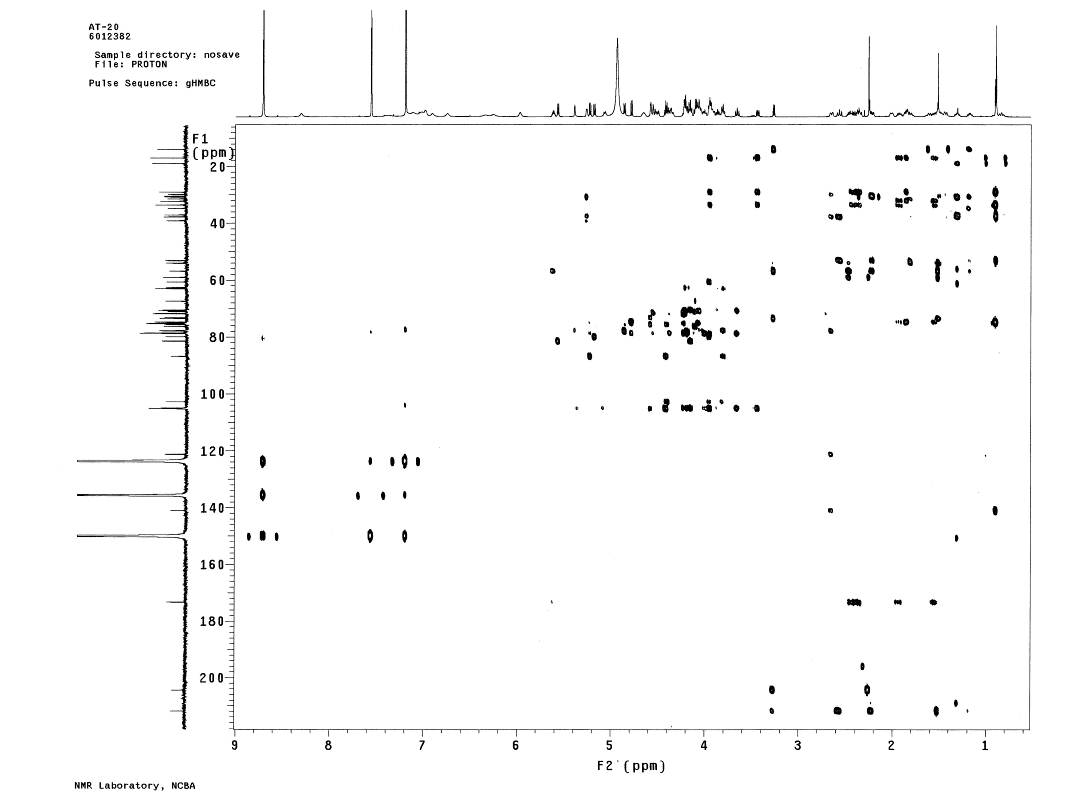


Figure Cf HMBC of compound **3**

Figure Da HR-ESI-MS of compound **4**


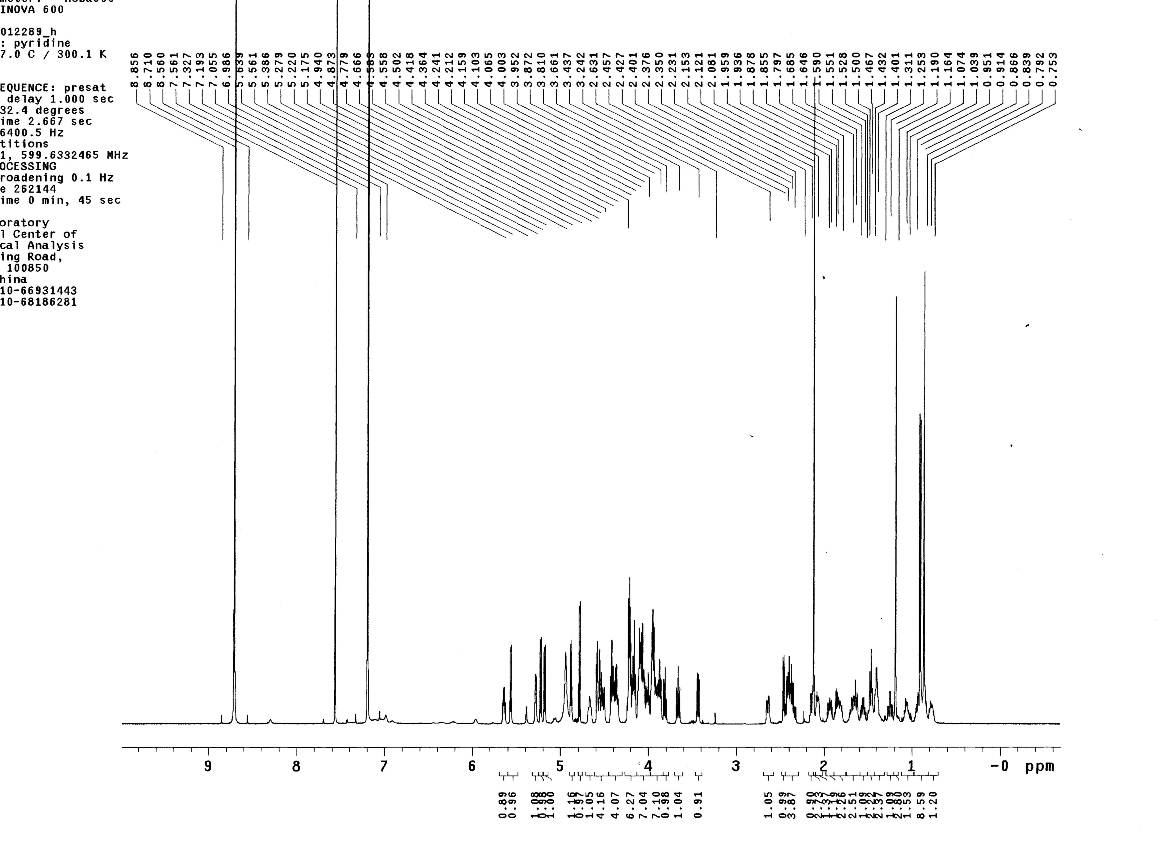


Figure Db ^1^H-NMR of compound **4**


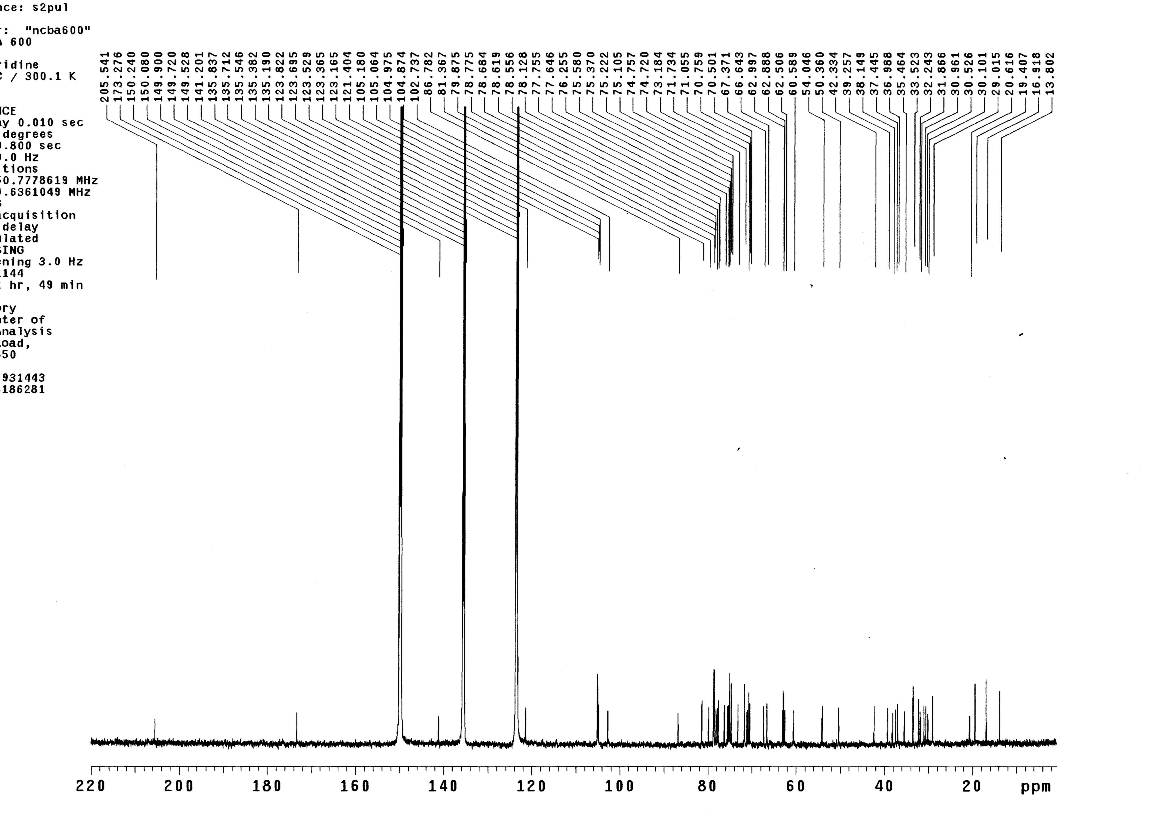


Figure Dc ^13^C-NMR of compound **4**


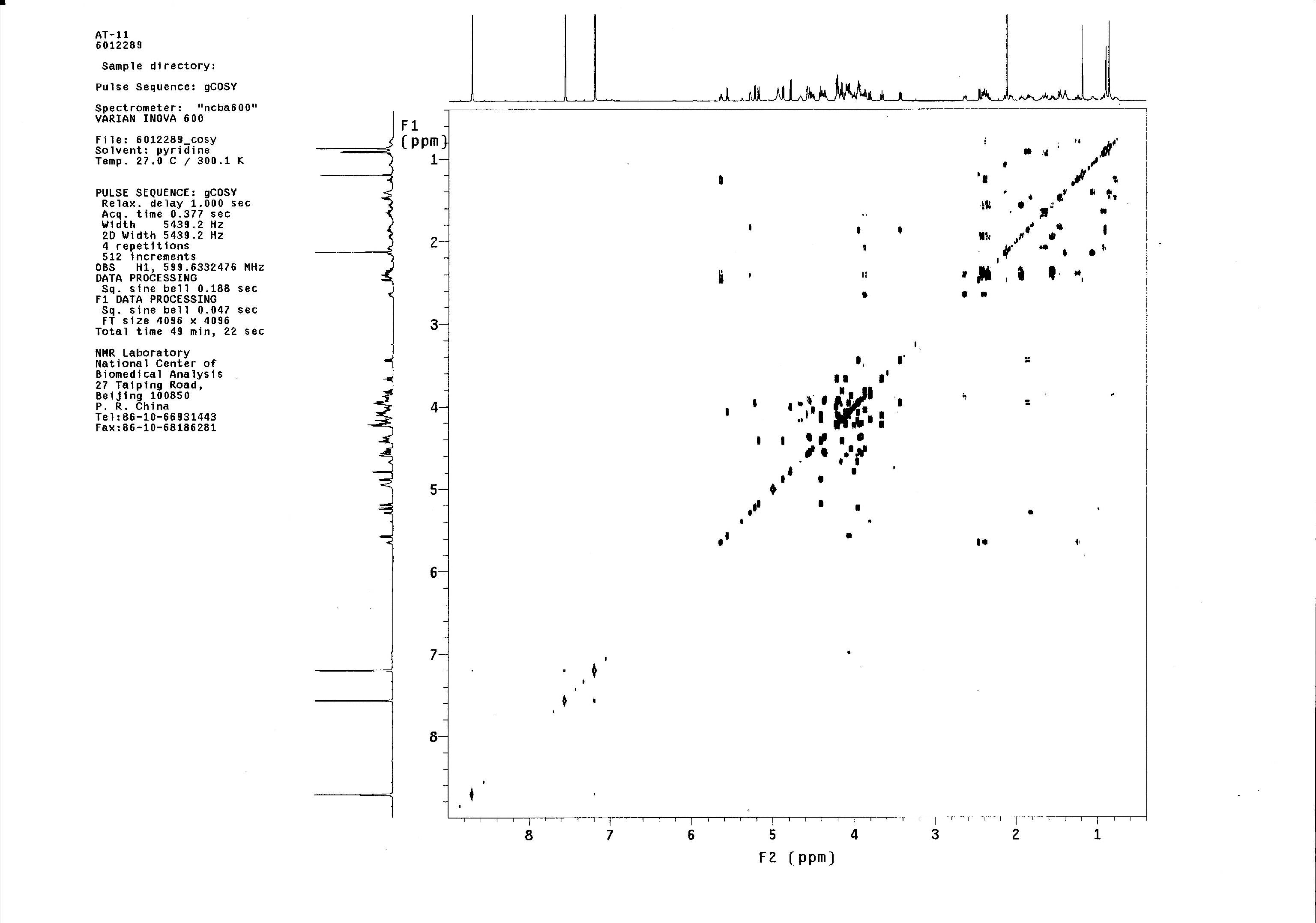


Figure Dd COSY of compound **4**


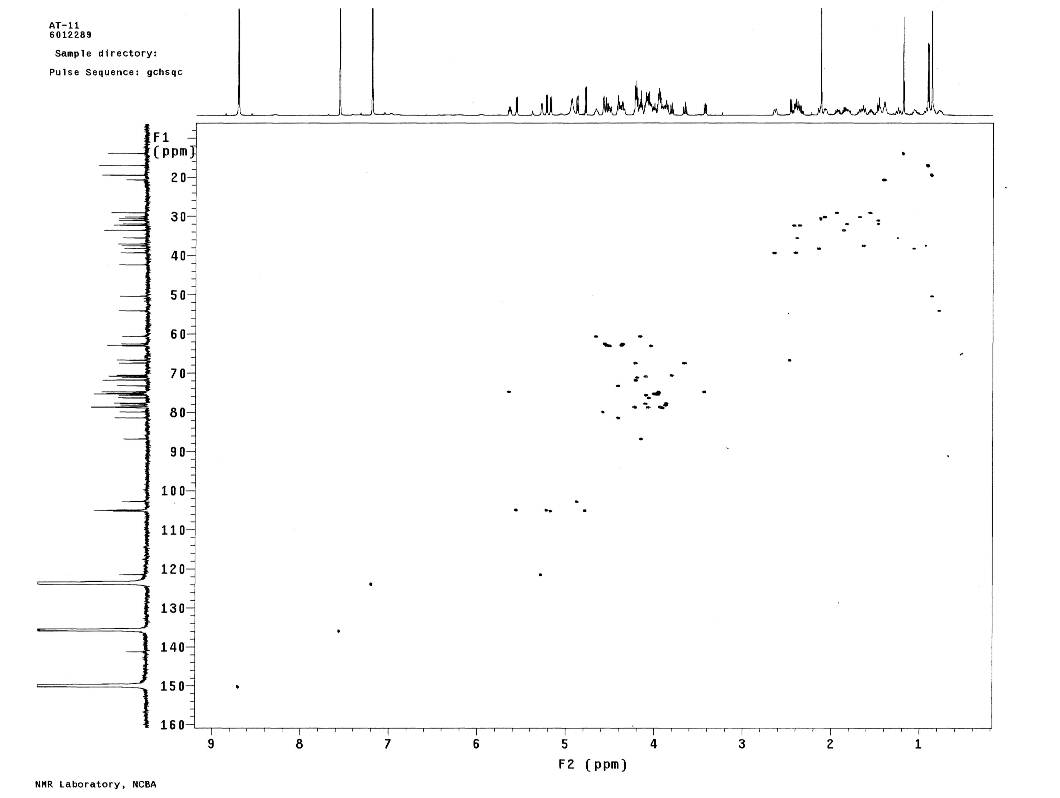


Figure De HSQC of compound **4**


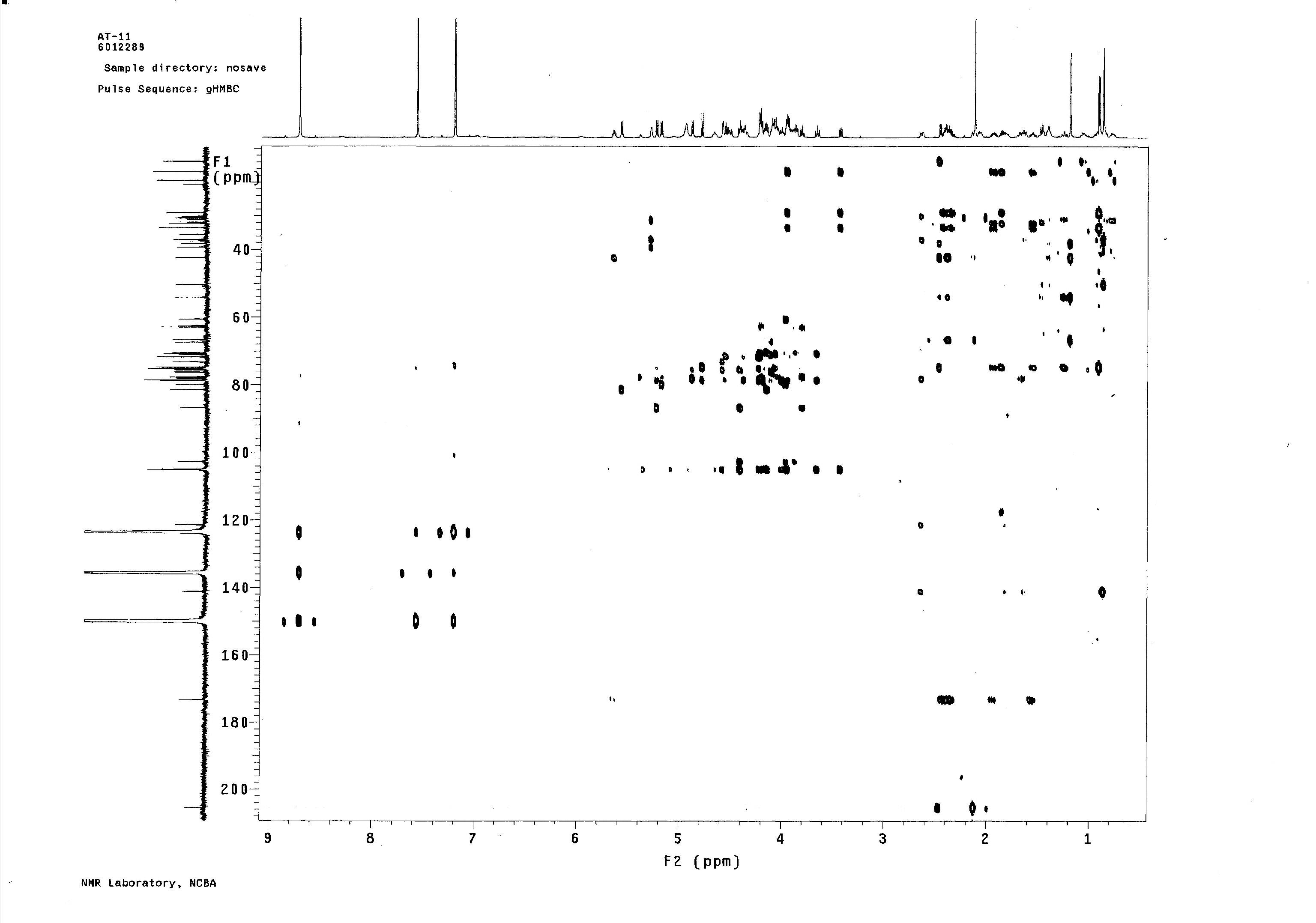


Figure Df HMBC of compound **4**

Figure Ea HR-ESI-MS of compound **5**


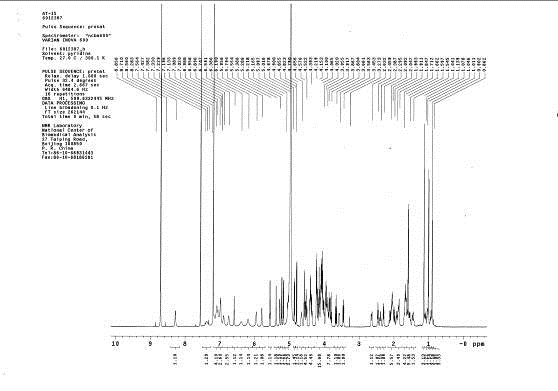
 Figure Eb ^1^H-NMR of compound **5**


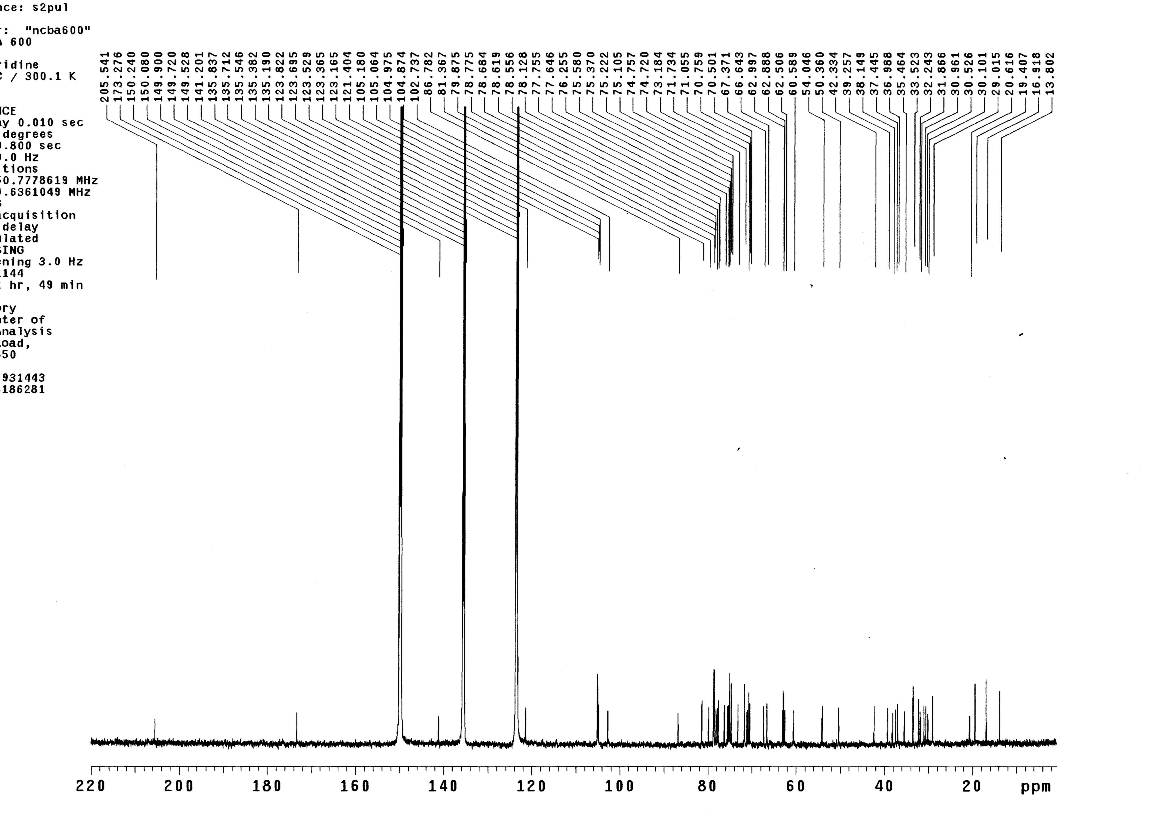


Figure Ec ^13^C-NMR of compound **5**

Figure Fa HR-ESI-MS of compound **6**


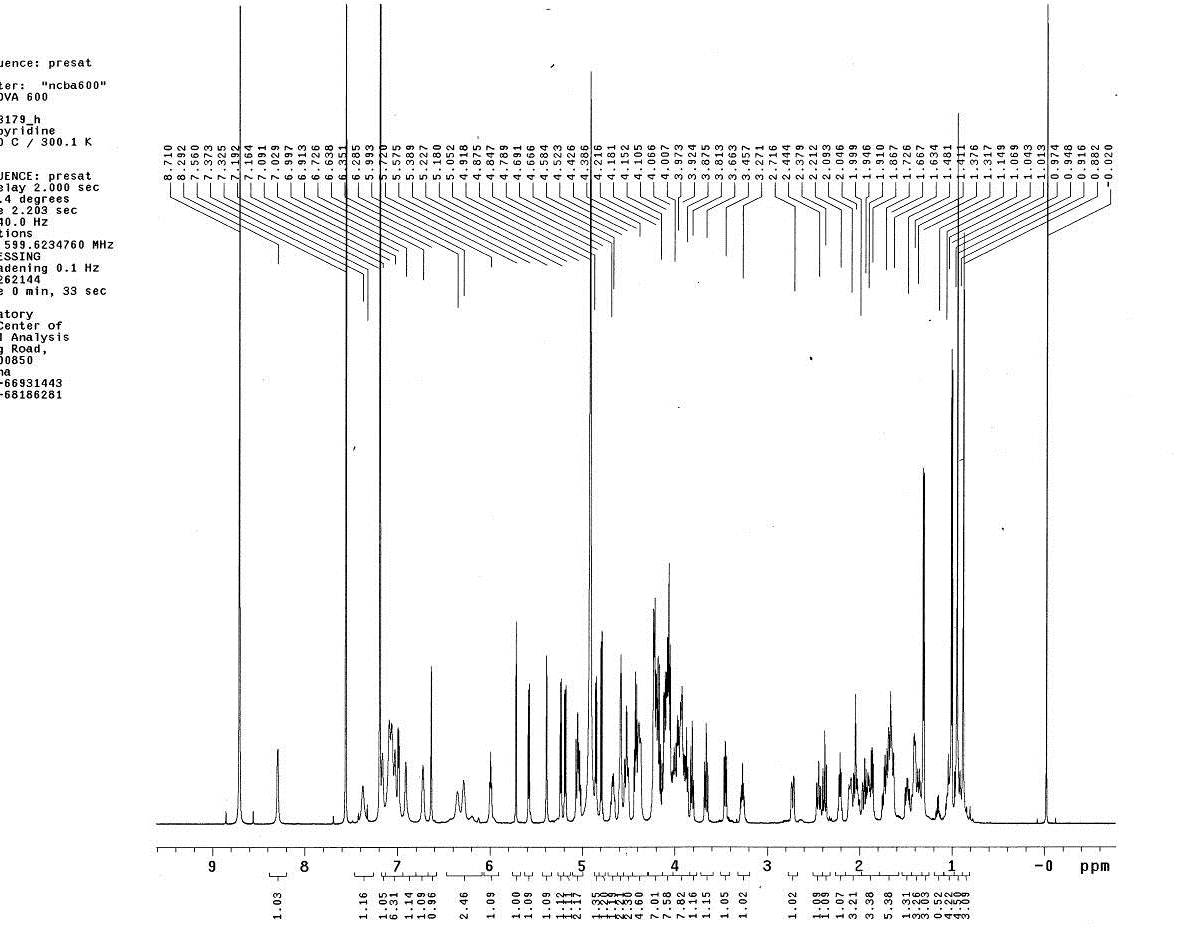


Figure Fb ^1^H-NMR of compound **6**


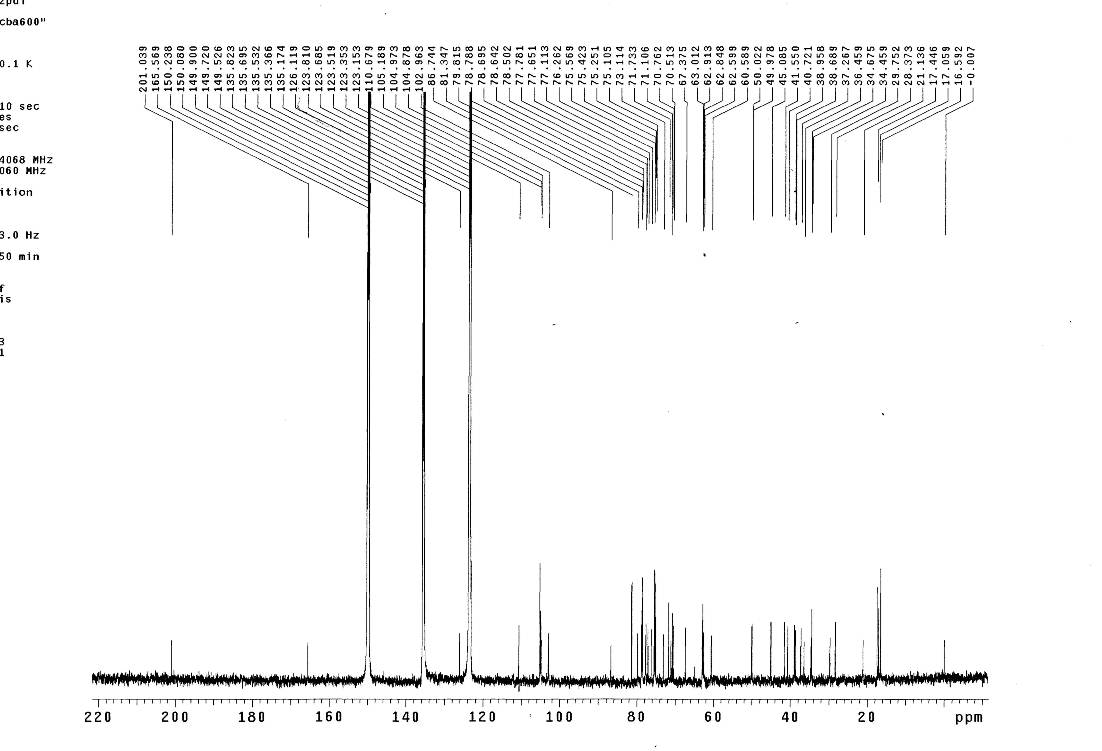


Figure Fc ^13^C-NMR of compound **6**


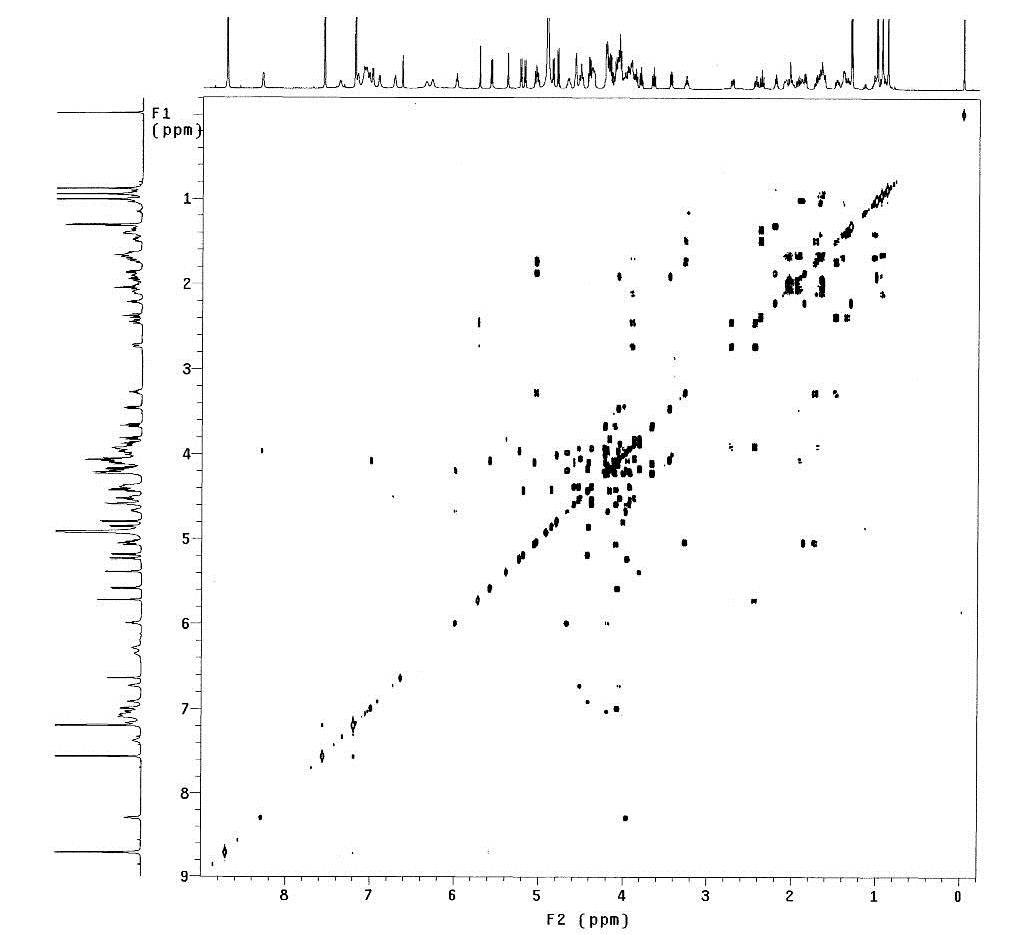


Figure Fd COSY of compound **6**


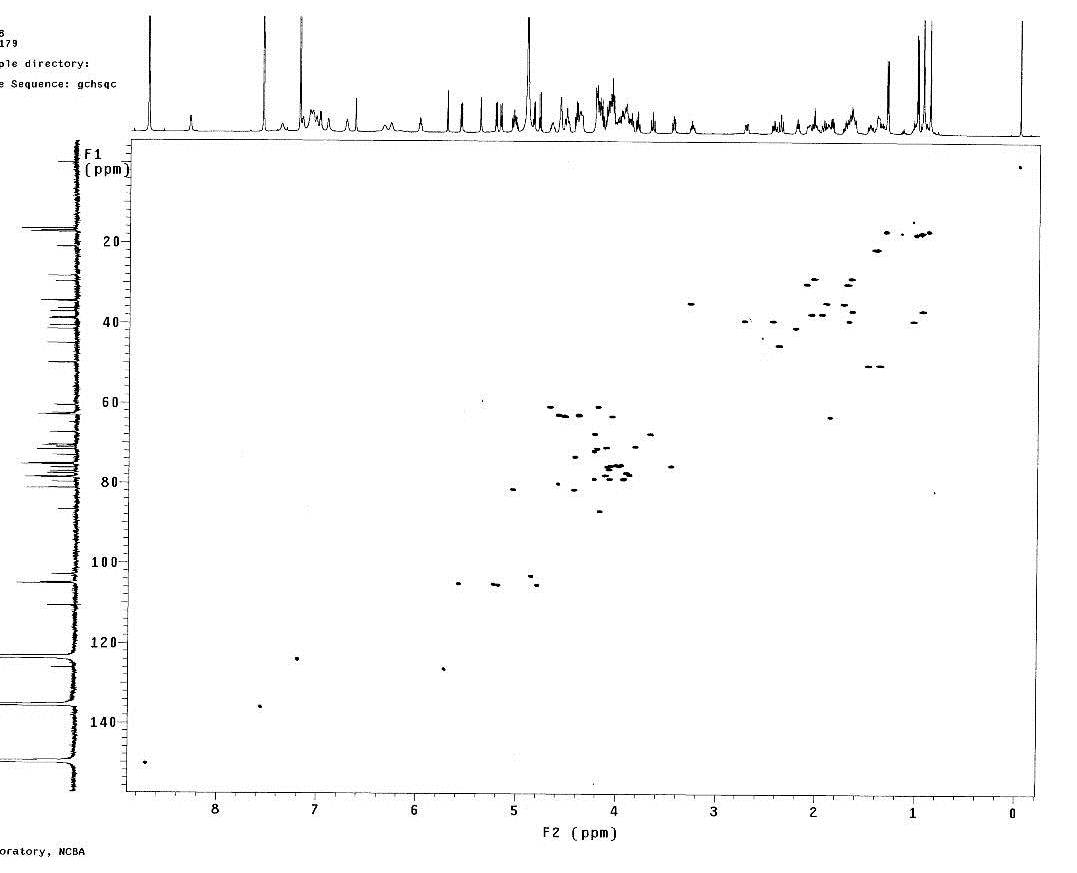


Figure Fe HSQC of compound **6**


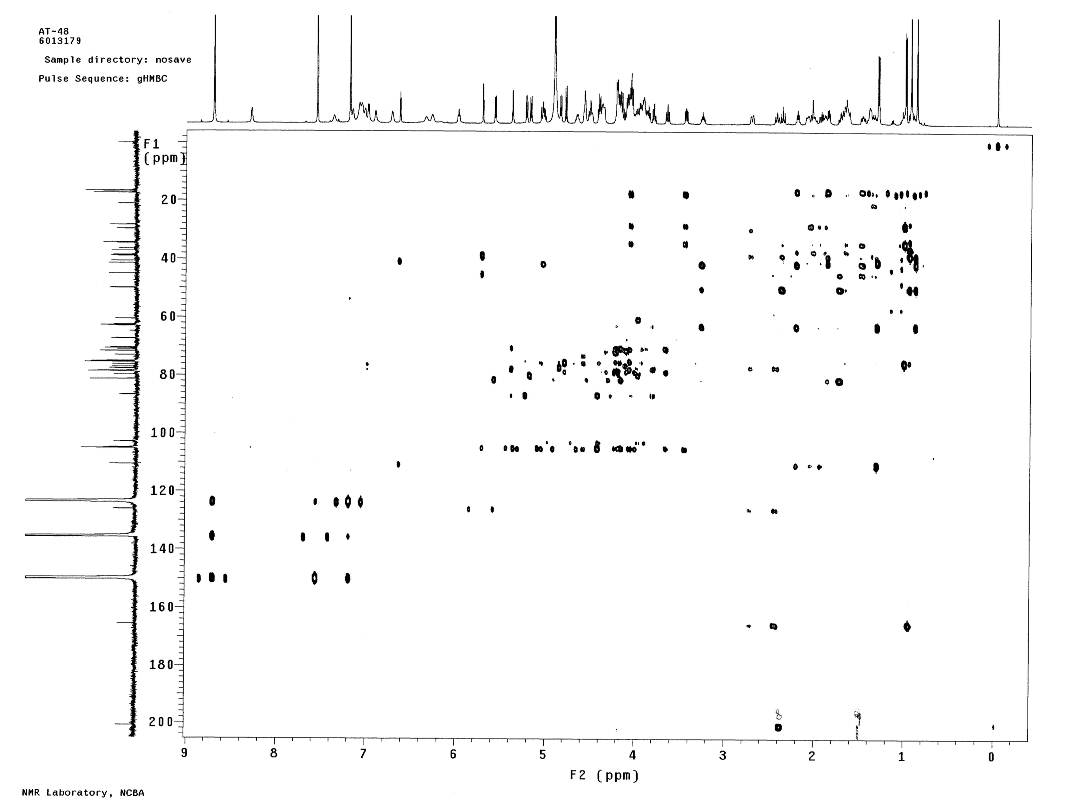


Figure Ff HMBC of compound **6**

Figure Ga HR-ESI-MS of compound **7**


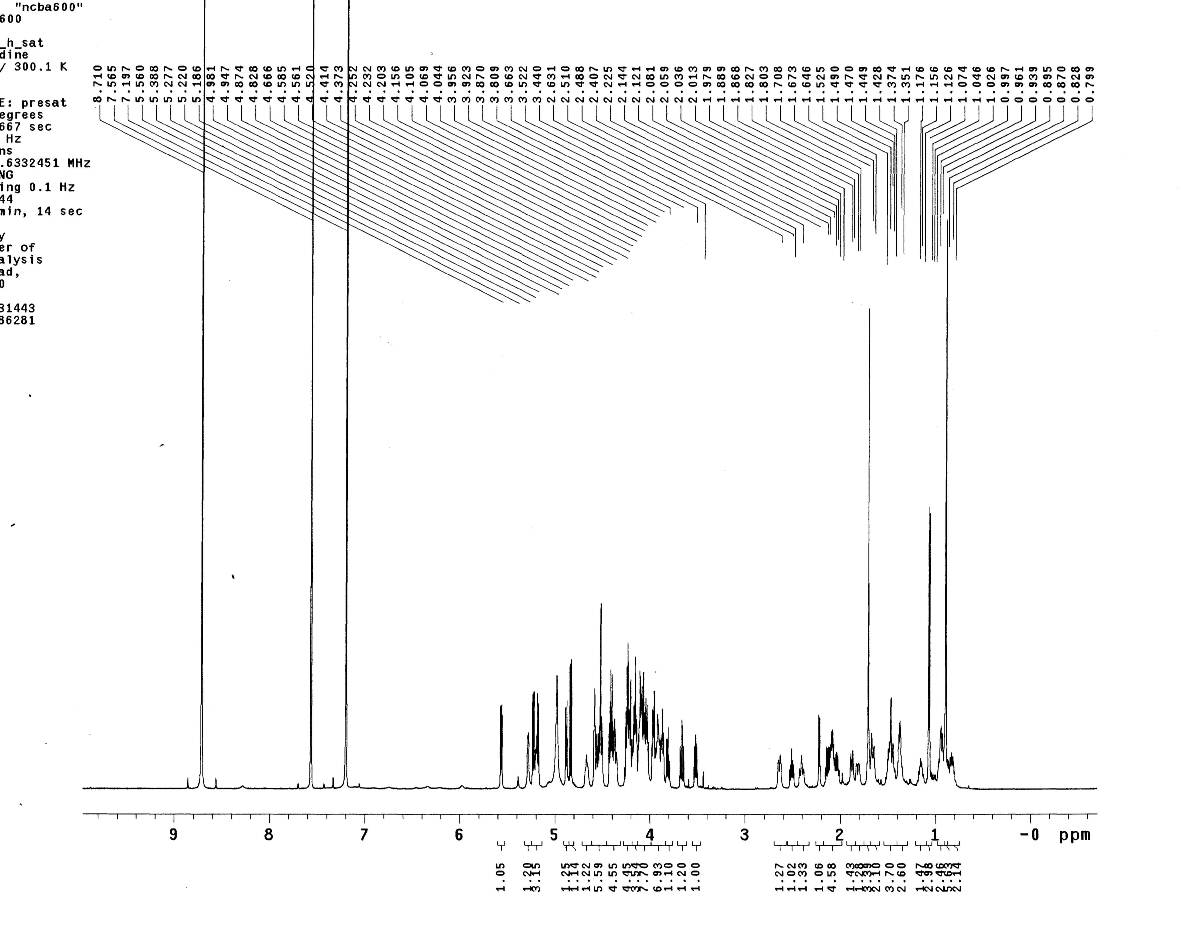


Figure Gb ^1^H-NMR of compound **7**


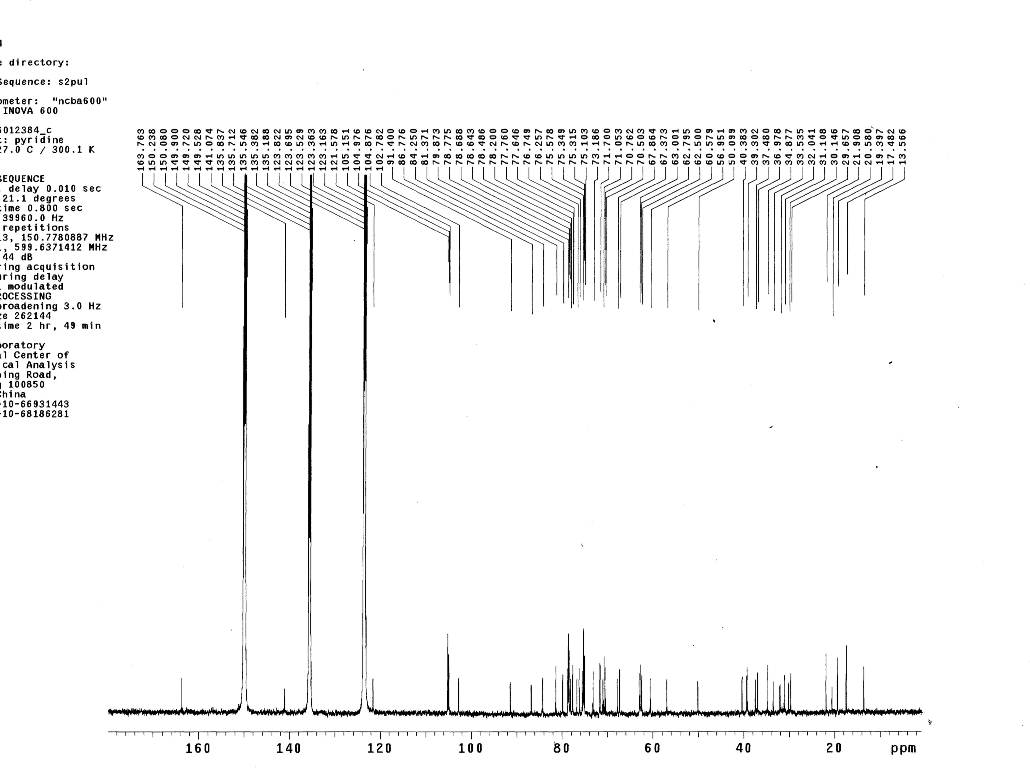


Figure Gc ^13^C-NMR of compound **7**


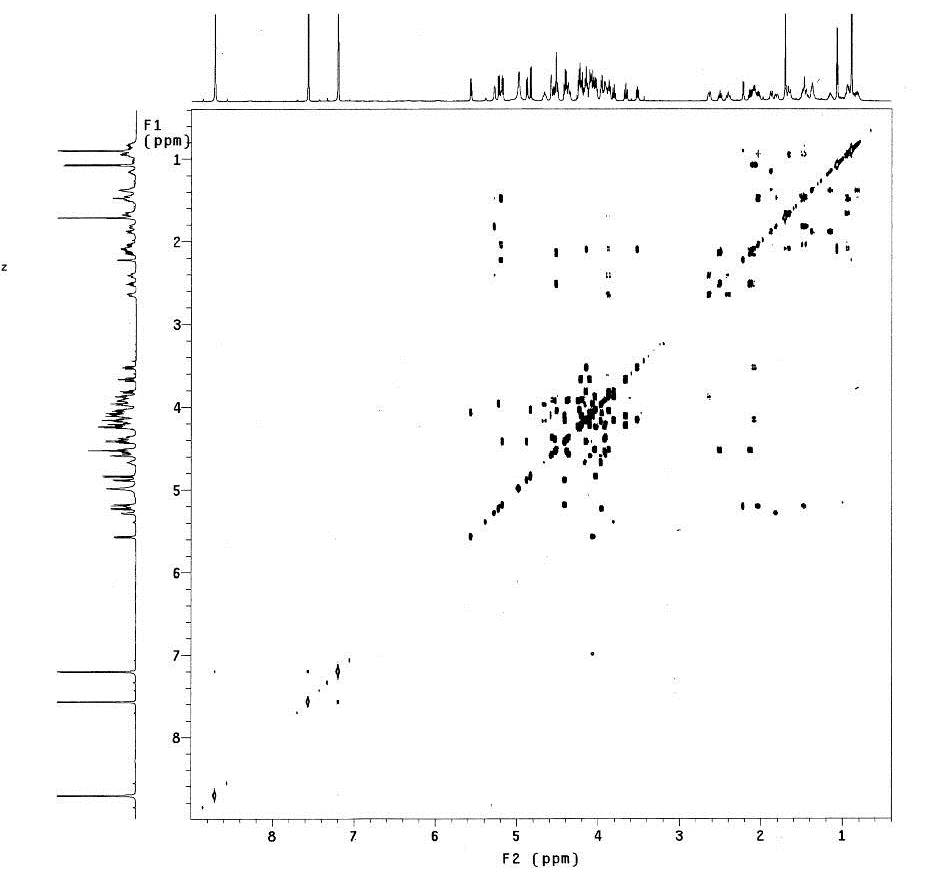


Figure Gd COSY of compound **7**


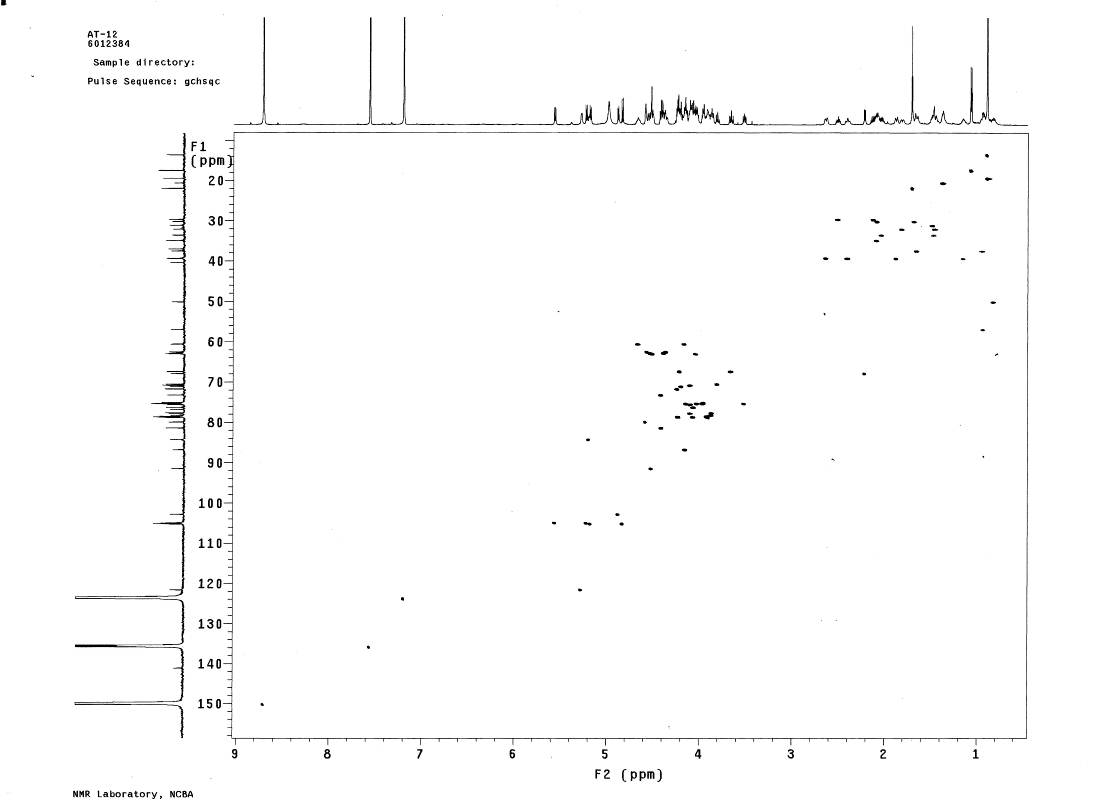


Figure Ge HSQC of compound **7**


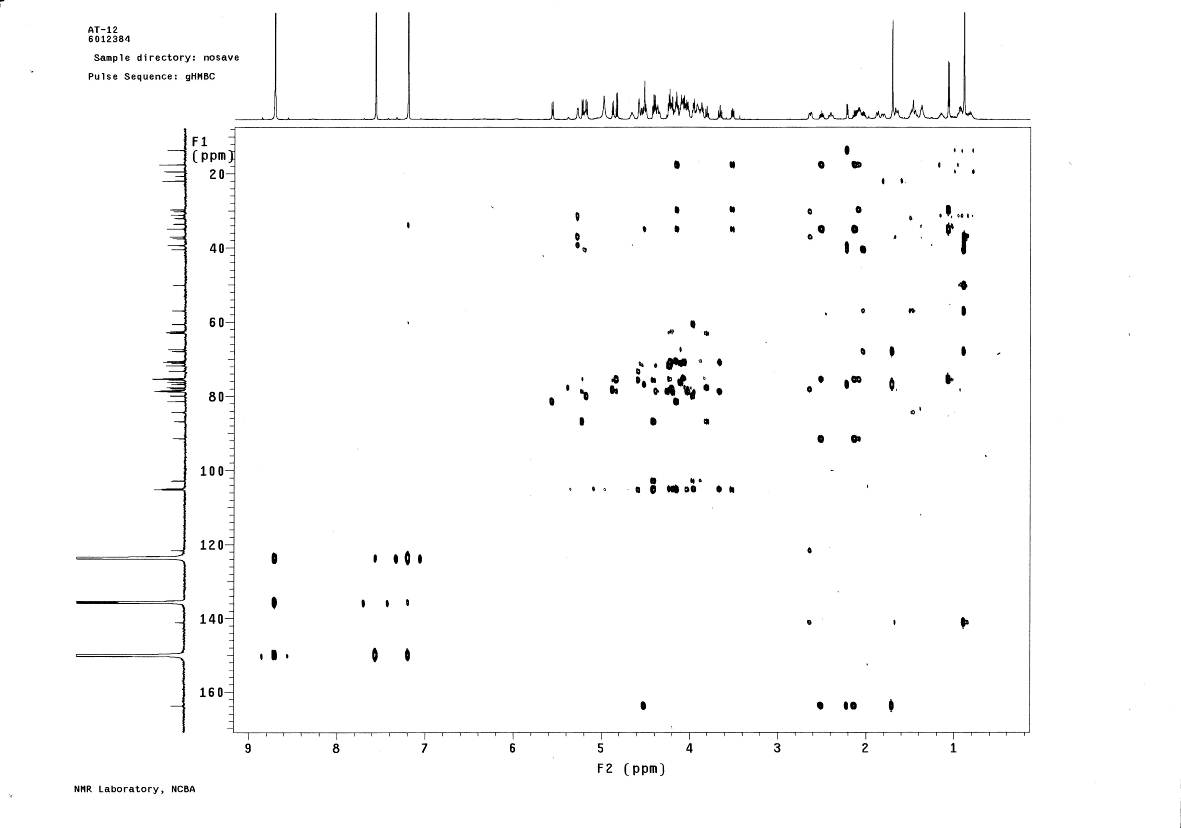


Figure Gf HMBC of compound **7**

**
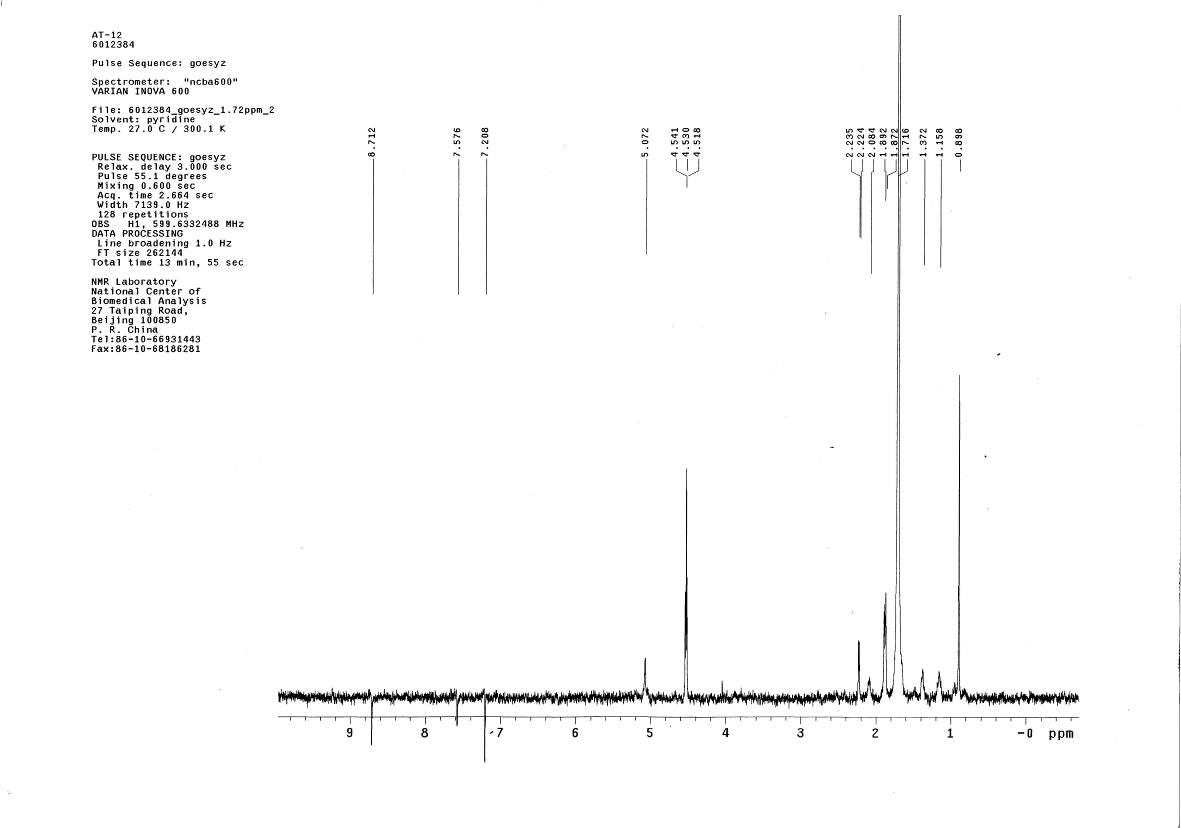
**

Figure Gg NOE of compound **7**

Figure Ha HR-ESI-MS of compound **8**


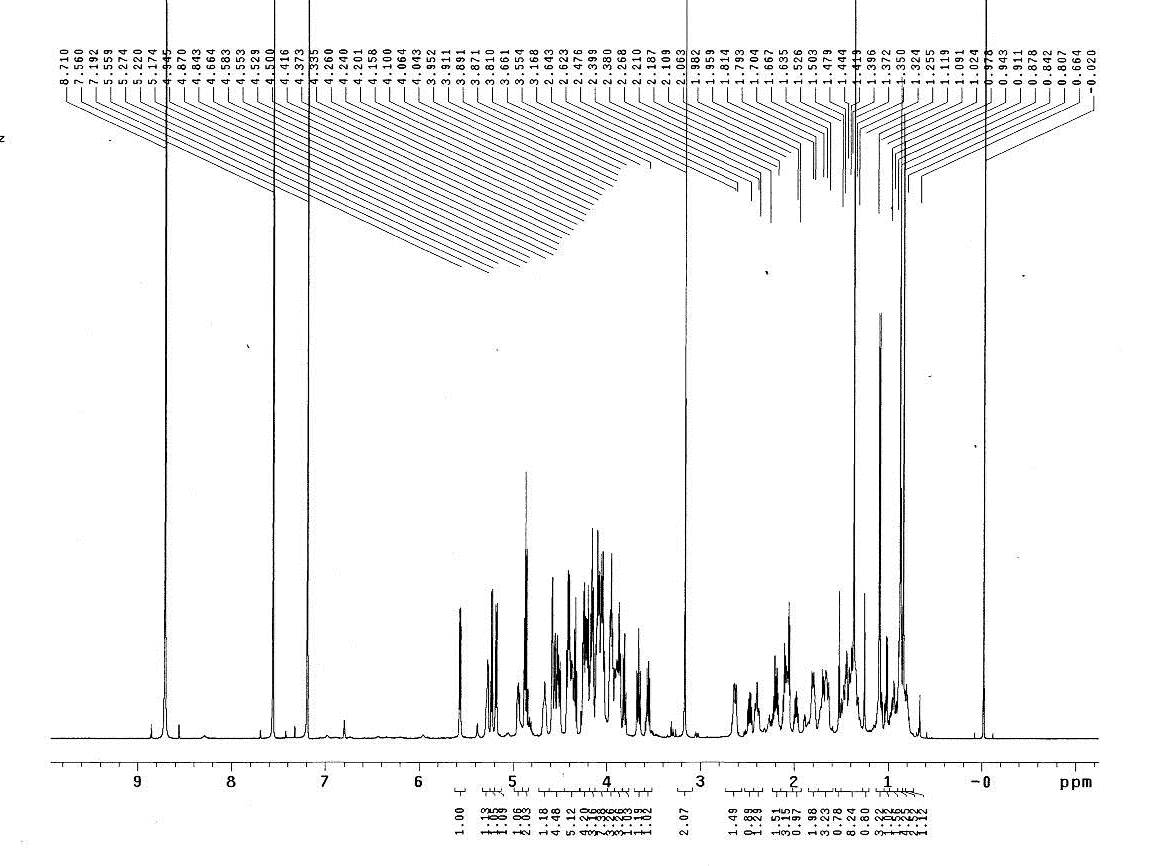


Figure Hb ^1^H-NMR of compound **8**


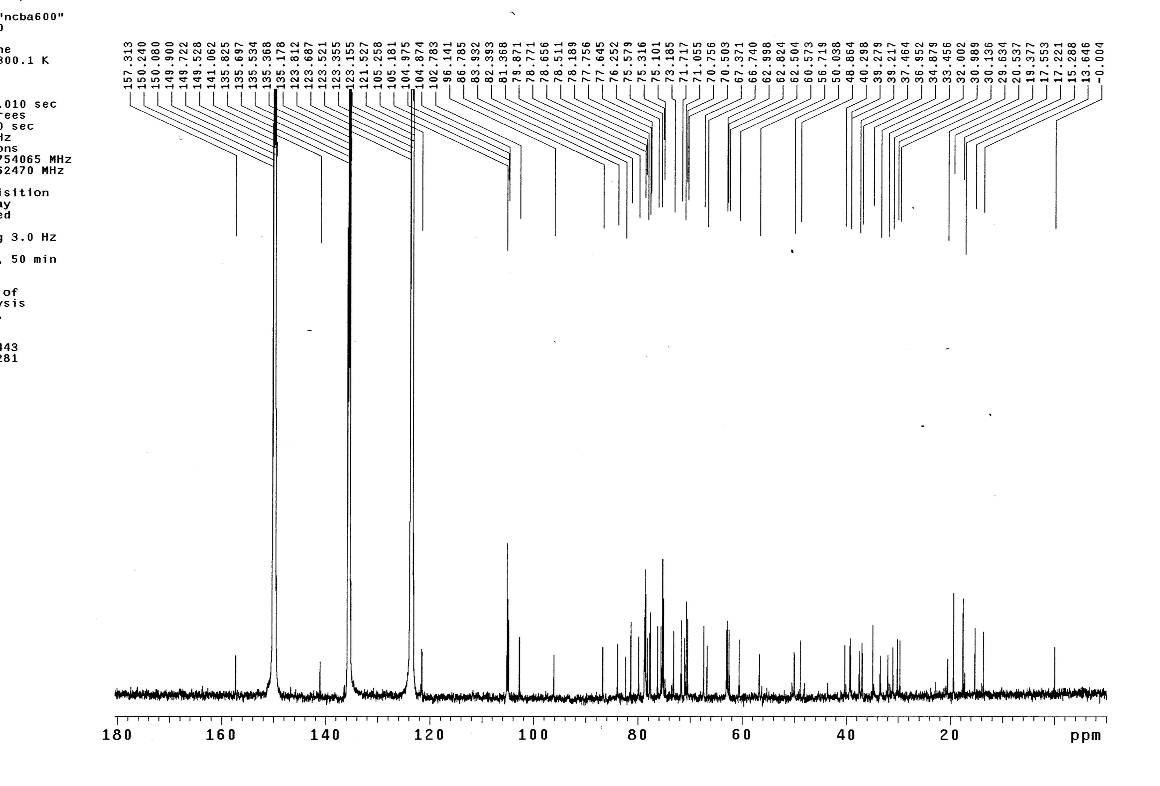


Figure Hc ^13^C-NMR of compound **8**

Figure Ia HR-ESI-MS of compound **9**


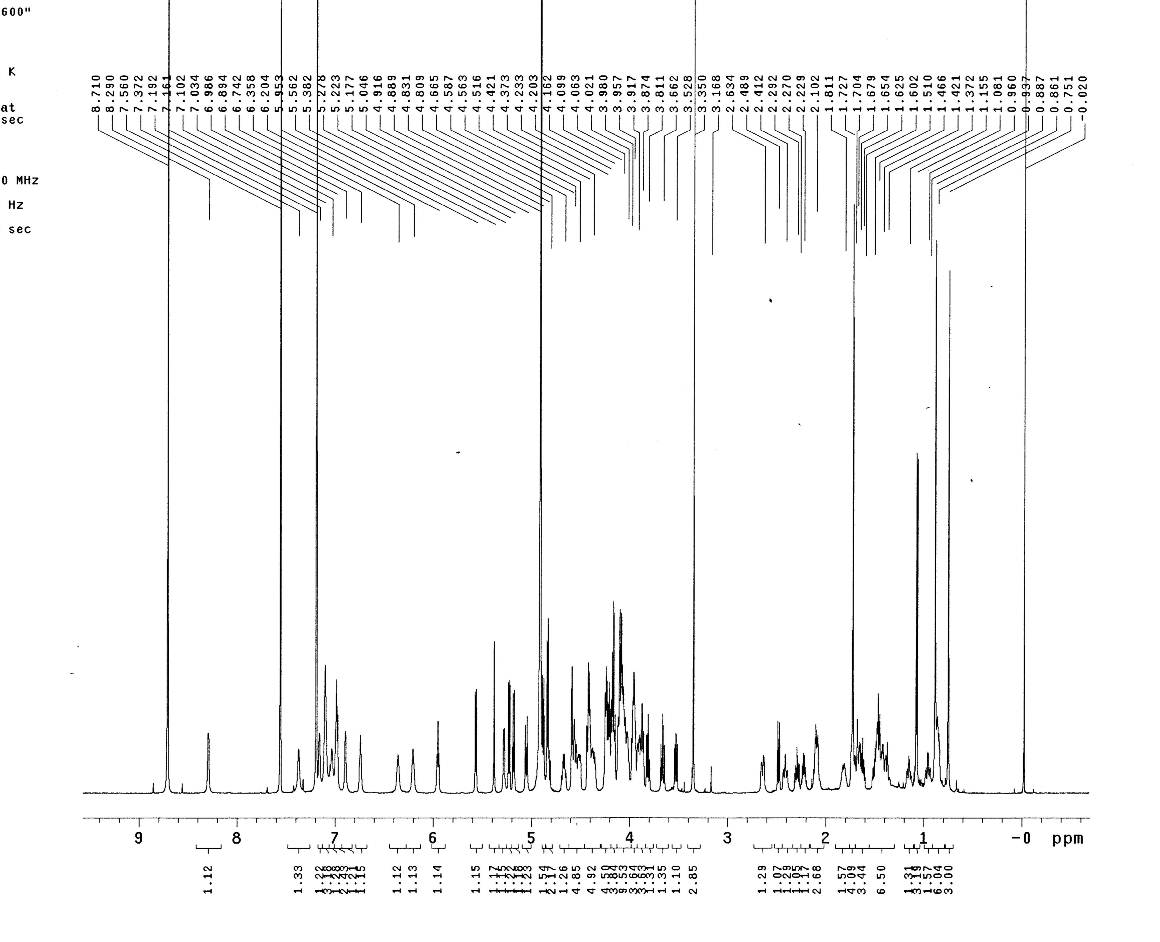


Figure Ib ^1^H-NMR of compound **9**


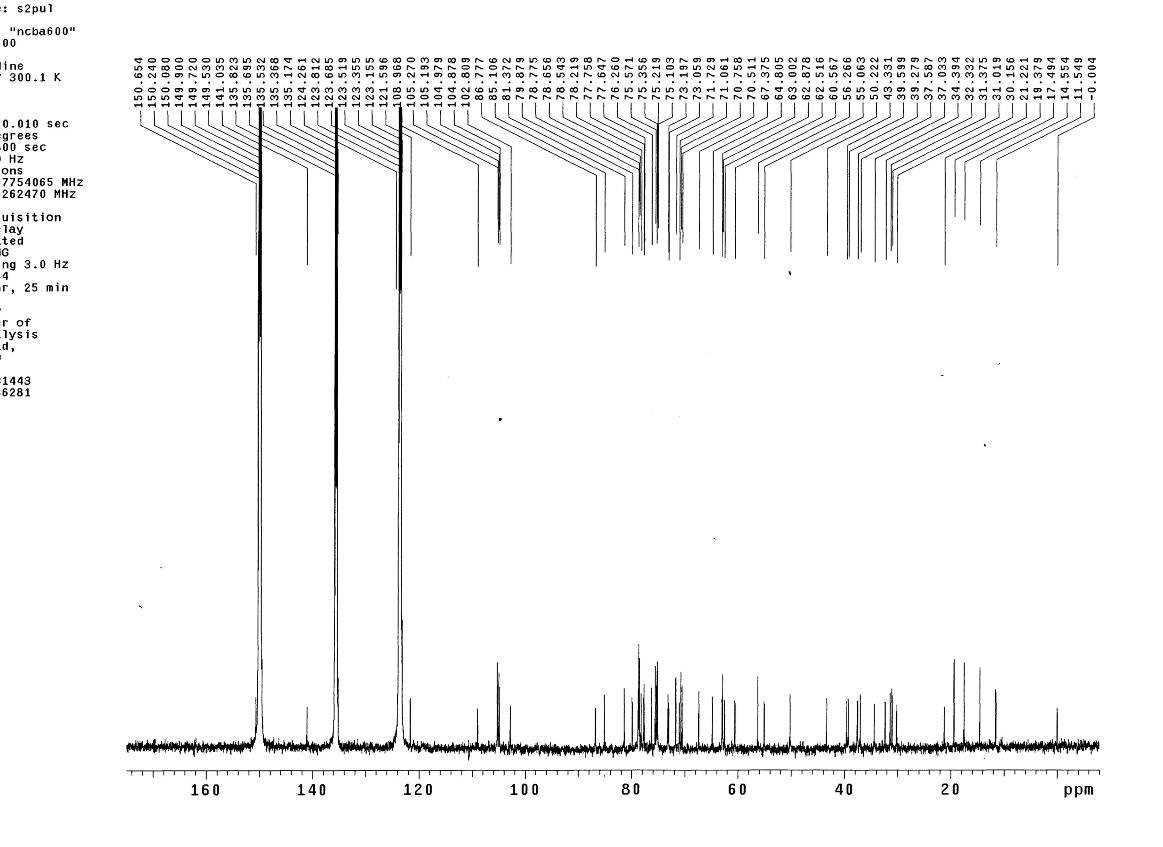


Figure Ic ^13^C-NMR of compound **9**

Figure Ja HR-ESI-MS of compound **10**


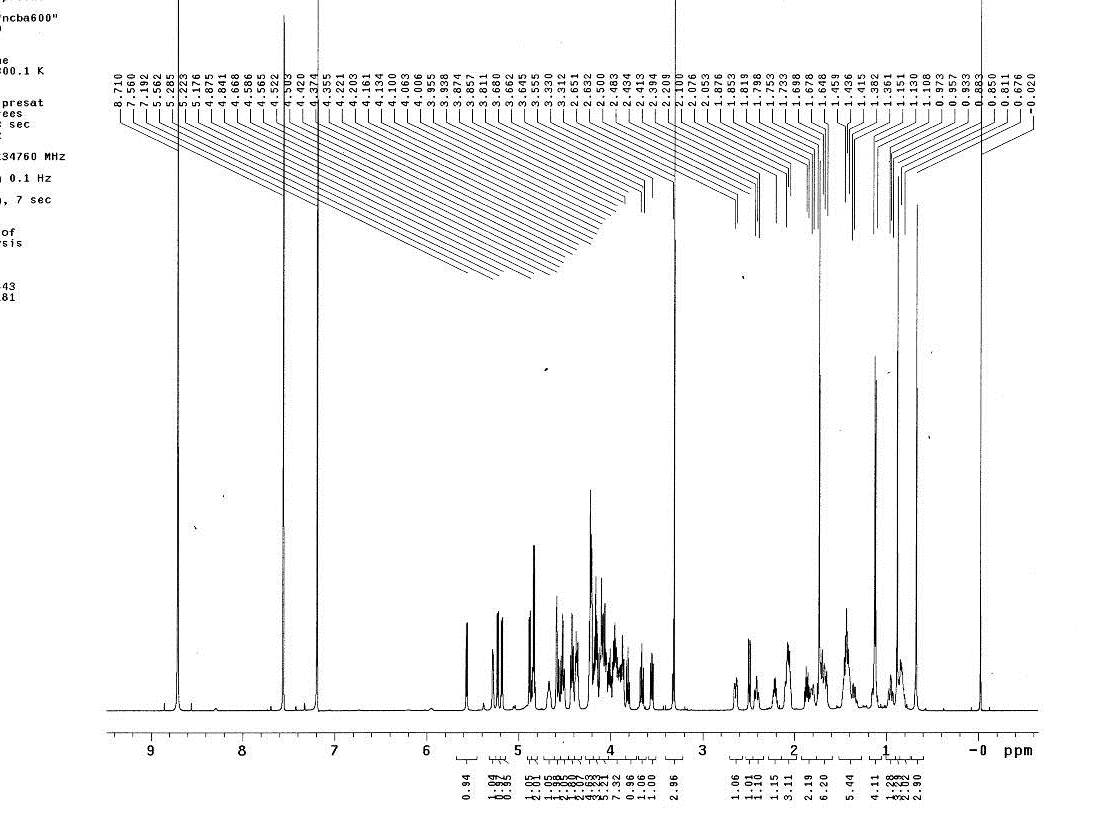


Figure Jb ^1^H-NMR of compound **10**


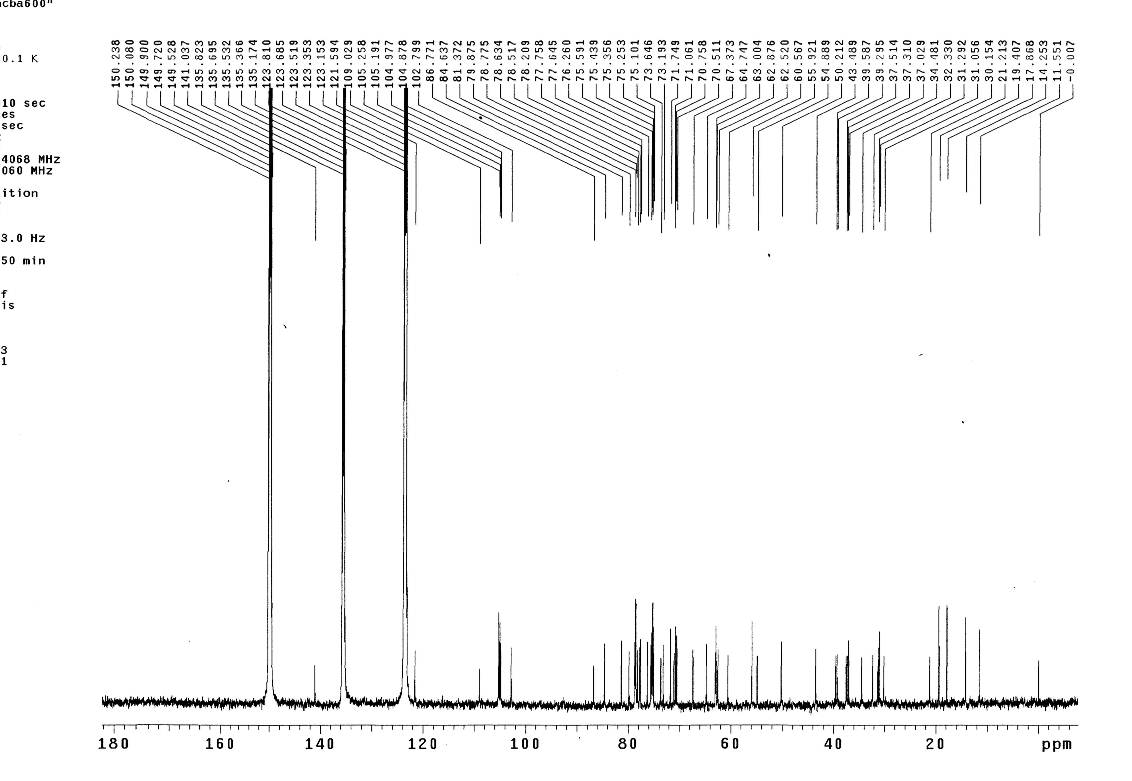
Figure Jc ^13^C-NMR of compound **10**

Figure Ka HR-ESI-MS of compound **11**


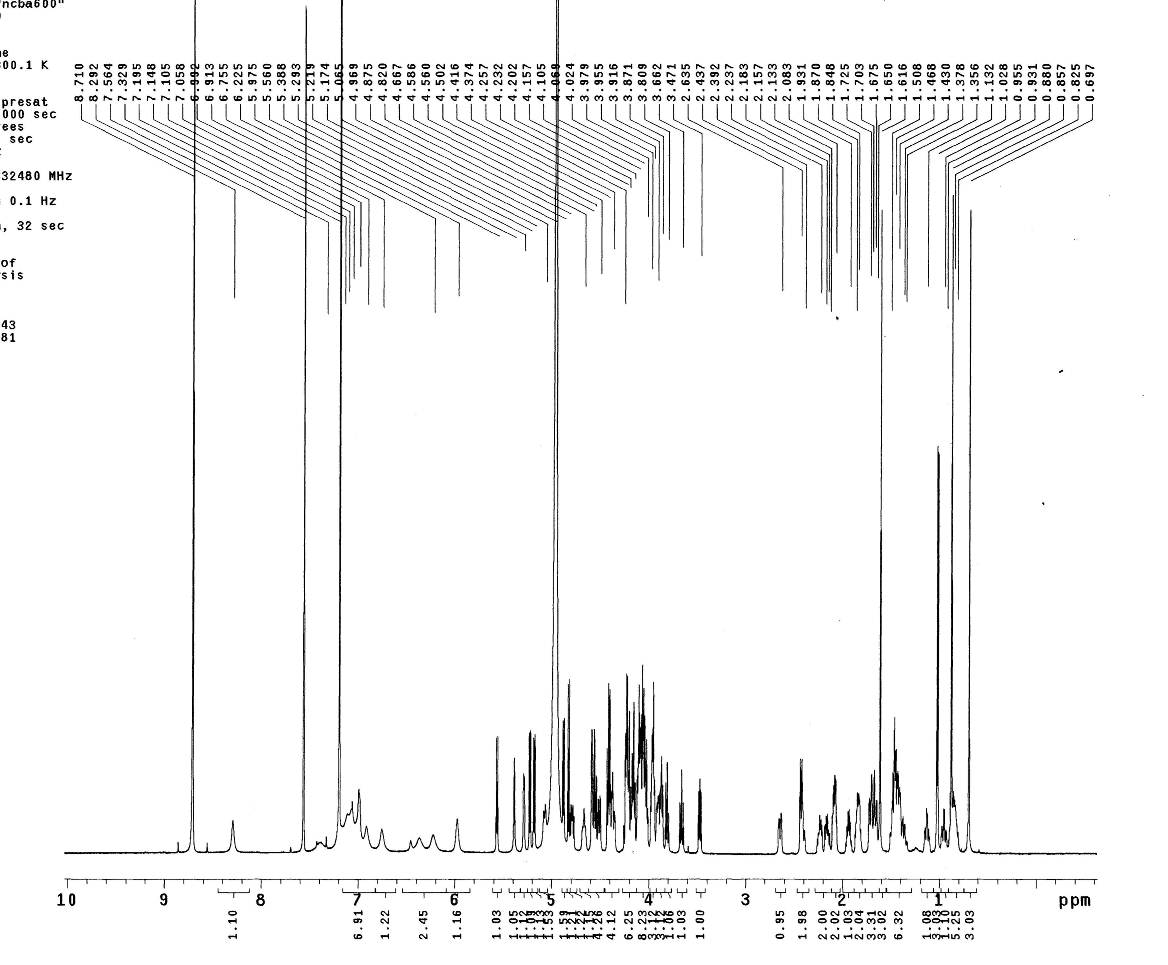


Figure Kb ^1^H-NMR of compound **11**


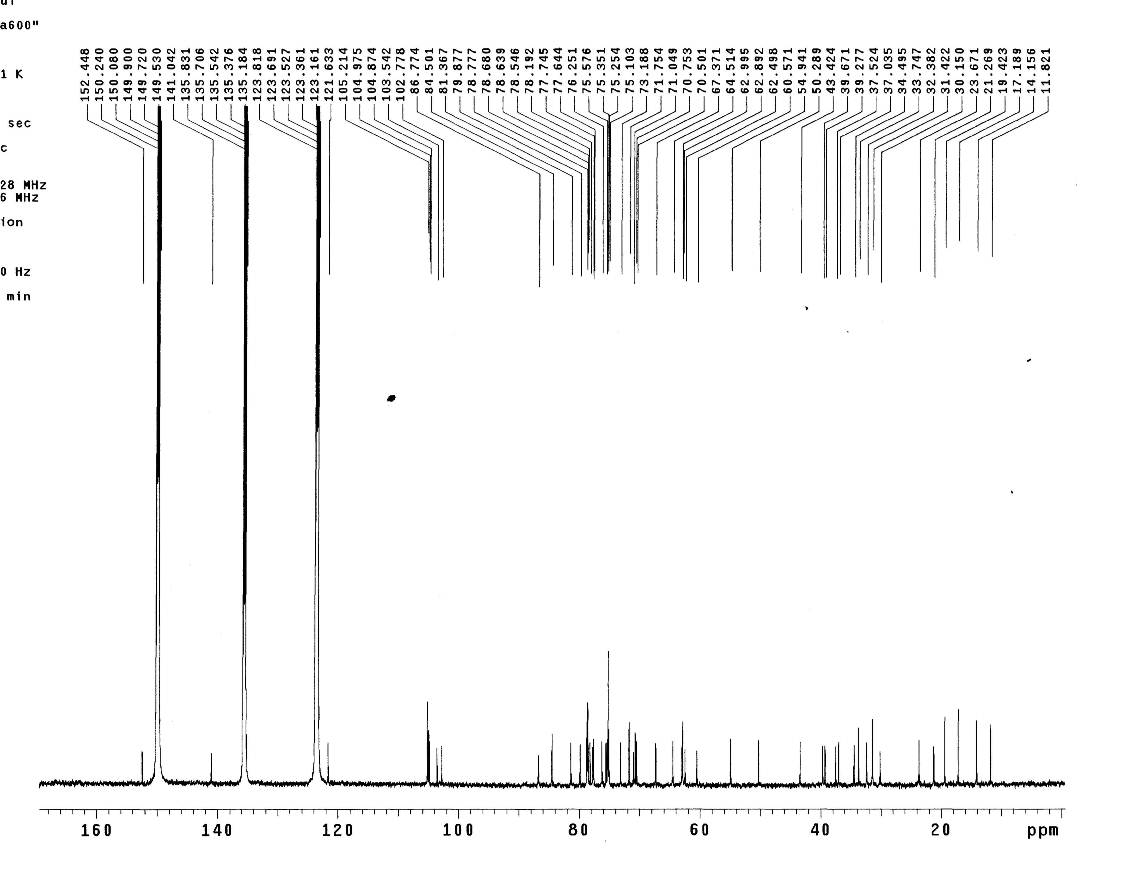


Figure Kc ^13^C-NMR of compound **11**

Figure La HR-ESI-MS of compound **12**


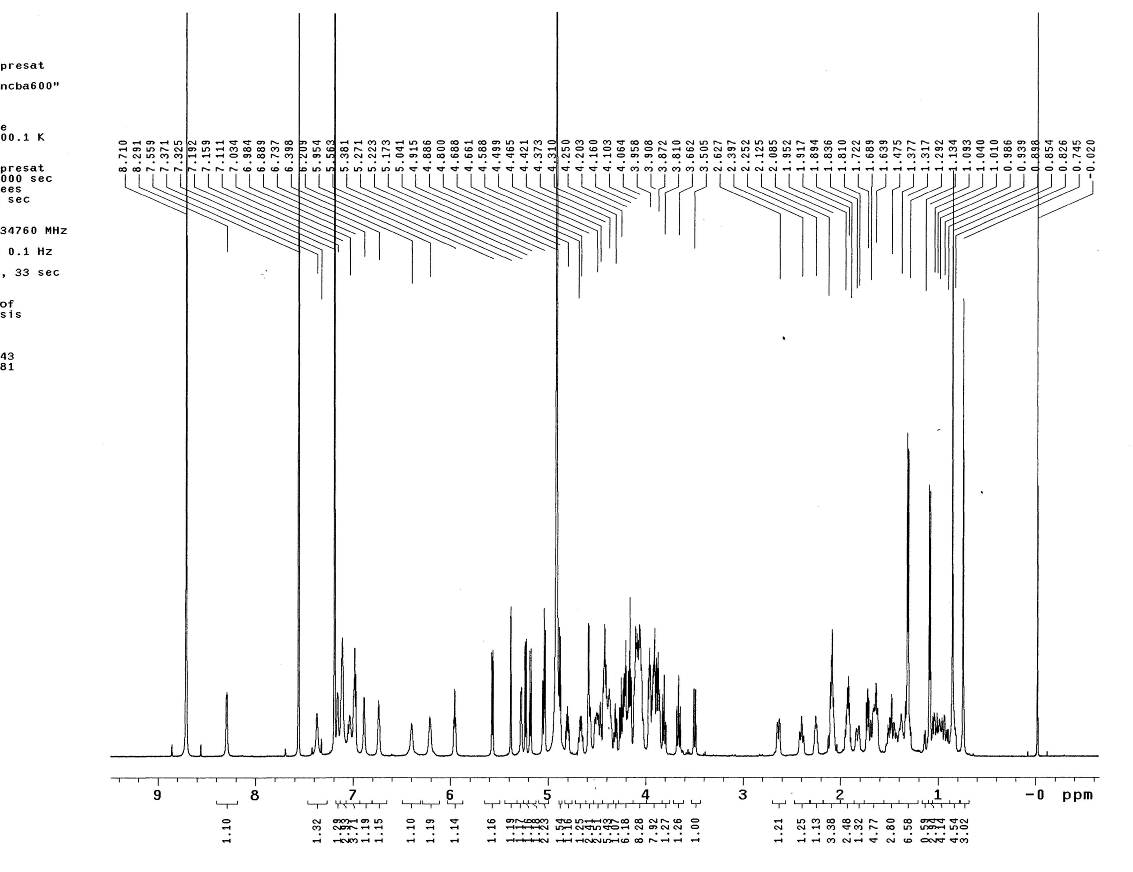


Figure Lb ^1^H-NMR of compound **12**


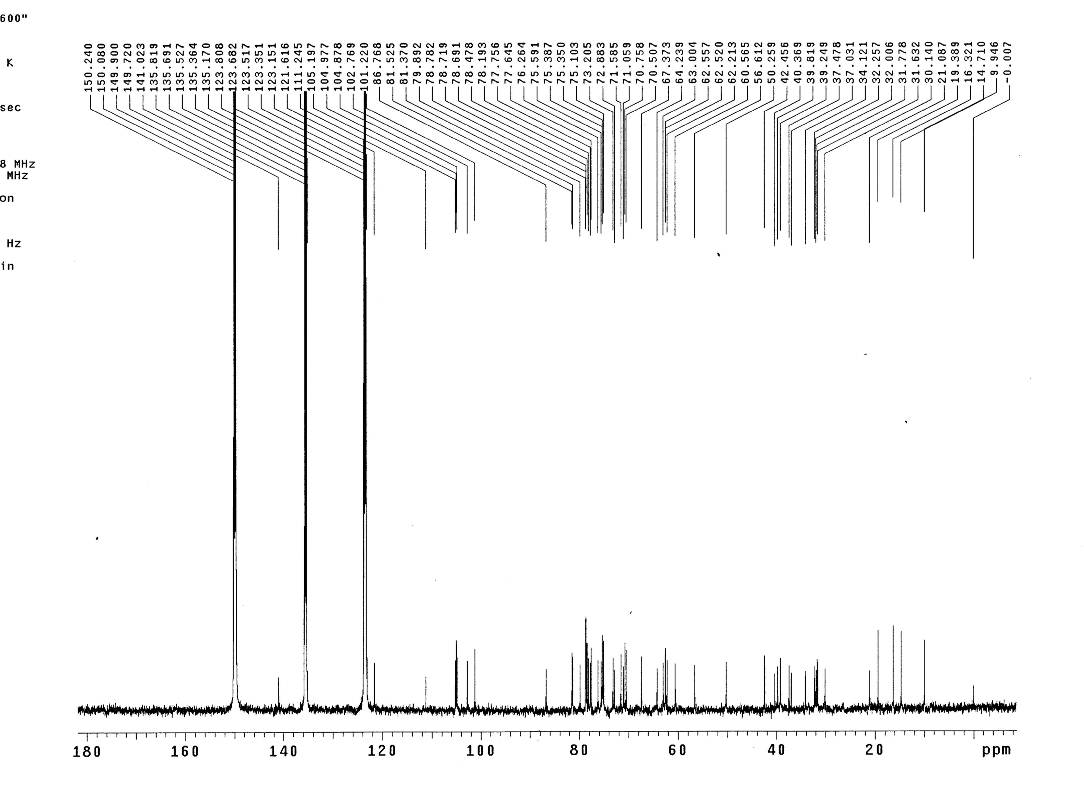


Figure Lc ^13^C-NMR of compound **12**

Figure Ld ^1^H-^1^H COSY of compound **12**

Figure Lc HSQC of compound **12**

Figure Ld HMBC of compound **12**
